# Convenient synthesis of the pentasaccharide repeating unit corresponding to the cell wall O-antigen of *Escherichia albertii* O4

**DOI:** 10.3762/bjoc.16.12

**Published:** 2020-01-22

**Authors:** Tapasi Manna, Arin Gucchait, Anup Kumar Misra

**Affiliations:** 1 Bose Institute, Division of Molecular Medicine, P-1/12, C.I.T. Scheme VII M, Kolkata 700054, India

**Keywords:** *Escherichia albertii* O4, glycosylation, HClO_4_/SiO_2_, O-antigen, pentasaccharide

## Abstract

A straightforward sequential synthetic strategy has been developed for the synthesis of a pentasaccharide repeating unit corresponding to the cell wall O-antigen of the *Escherichia albertii* O4 strain in very good yield with the desired configuration at the glycosidic linkages using thioglycosides and trichloroacetimidate derivatives as glycosyl donors and perchloric acid supported over silica (HClO_4_/SiO_2_) as a solid supported protic acid glycosyl activator. The expected configuration at the glycosidic linkages was achieved using a reasonable selection of protecting groups in the manosaccharide intermediates.

## Introduction

**Figure 1:**
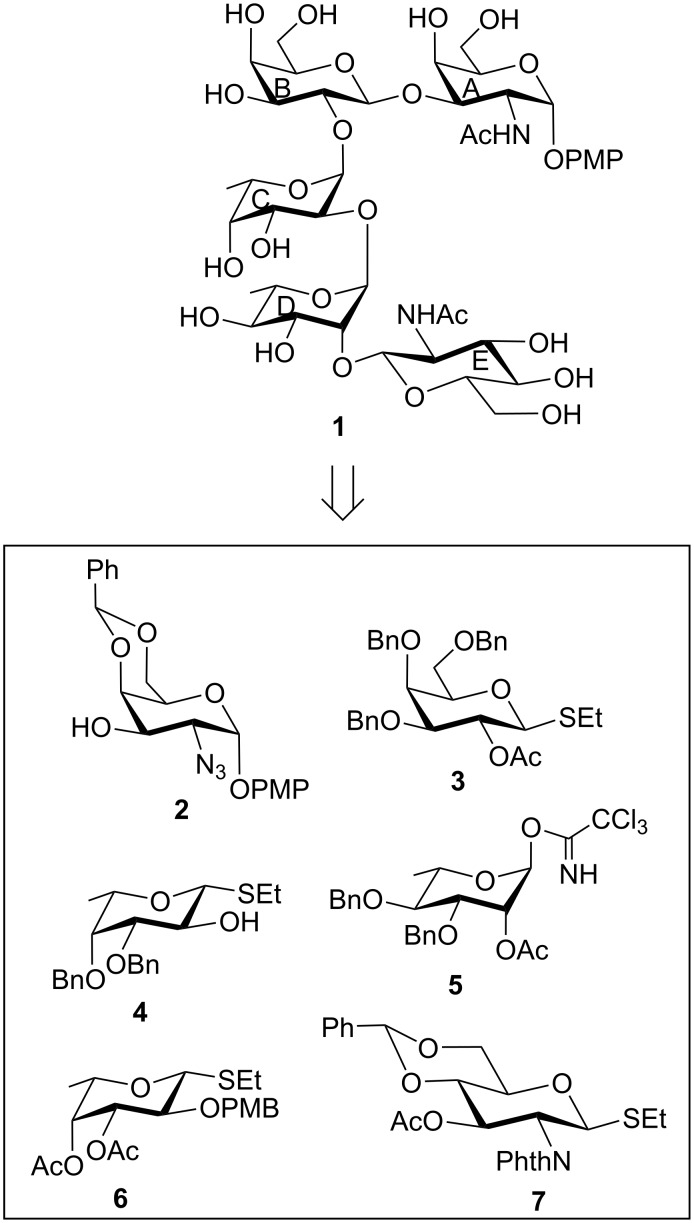
Structure of the pentasaccharide repeating unit corresponding to the cell wall O-antigen of *Escherichia albertii* O4 and its synthetic intermediates.

Diarrheal outbreaks are serious concerns all over the world particularly in the developing countries due to inadequate sanitation systems [1]. In most of the cases, the enteric infections originated due to the intake of less cooked food and contaminated water [2]. Several strains of *Shigella* [3], *Salmonella* [4] and enteropathogenic *Escherichia coli* [5] are commonly known for causing diarrheal infections. Besides the mainstream enteropathogenic bacterium, *Escherichia albertii* (*E. albertii*) is an emerging human pathogen causing gastroenteric infections in different countries [6]. Although, this species was identified earlier as *Hafnia alvei*, later it was redesignated as *E. albertii* [7]. *E. albertii* acted as a causative agent for diarrheal diseases in children with vomiting, fever and abdominal distension [8]. Several strains of *E. albertii* have been identified till date, which significantly contributed to the spreading of devastating diarrheal infections in different countries [9]. The role of cell wall O-polysaccharides in regulating the virulence properties of bacteria is well established [10]. Recently, Naumenko et al. [11] reported the structure of the repeating unit of the cell wall O-polysaccharide of the *E. albertii* O4 strain [11], which is a pentasaccharide comprising of α-linked ᴅ-galactosamine, β-linked ᴅ-glucosamine, β-linked ᴅ-galactose, α-linked ʟ-fucose and α-linked ʟ-rhamnose moieties. In the recent past, several vaccine candidates have been developed to control bacterial infections by conjugating cell wall polysaccharides with suitable proteins, which include vaccines against *Haemophilia influenza* type b (Hib) [12–13], meningitis [14], pneumococcal infections [15–16] and enteric diseases such as cholera [17], diarrhea [18] and urinary tract infections [19]. Despite the possibility of isolating the polysaccharides by fermentation techniques, it is difficult to get a significant quantity of polysaccharide fragments from natural sources with adequate purity. Therefore, the development of chemical synthetic strategies is quite pertinent to obtain a requisite quantity of oligosaccharide fragments with adequate purity. In this direction, the total synthesis of the pentasaccharide repeating unit corresponding to the cell wall O-antigenic polysaccharide of the *E. albertii* O4 strain using a sequential glycosylation strategy is presented herein (Figure 1).

## Results and Discussion

**Scheme 1:**
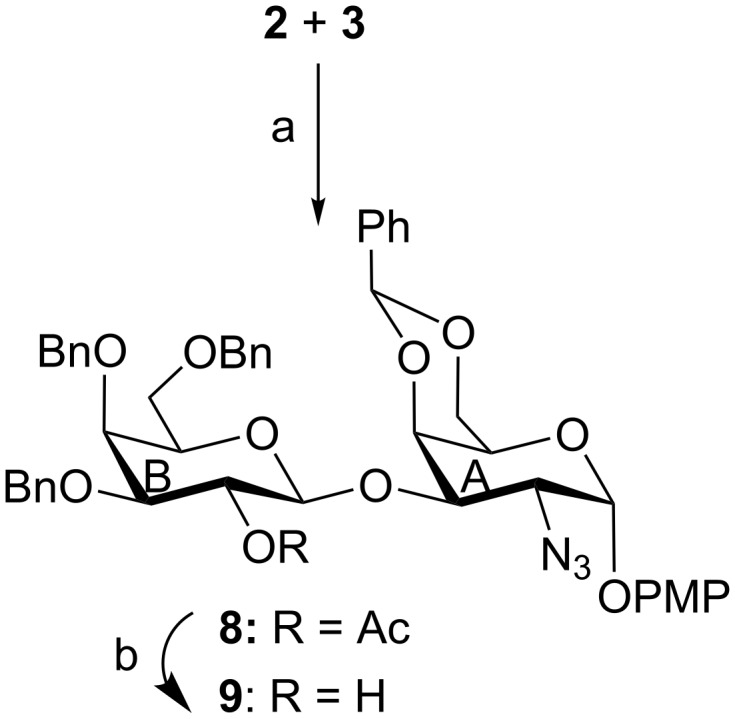
(a) NIS, HClO_4_/SiO_2_, MS 4 Å, CH_2_Cl_2_, −45 °C, 1 h, 79%; (b) 0.1 M CH_3_ONa, CH_3_OH, room temperature, 2 h, 95%.

The synthesis of pentasaccharide **1** was achieved using a convergent as well as a block synthetic strategy. For this purpose, a series of suitably functionalized monosaccharide intermediates **2** [20], **3** [21], **4** [22], **5** [23], **6** [24] and **7** [25] were prepared from the commercially available reducing sugars utilizing the reaction conditions reported in the literature (Figure 1). Although the monosaccharide intermediates used for the construction of the pentasaccharide derivative **15** are known in the literature, preparation of these intermediates required multiple step reaction sequences. Having obtained the monosaccharide intermediates, it was decided to proceed through a step-economic block synthetic strategy to achieve the target pentasaccharide derivative. Accordingly, stereoselective glycosylation of a ᴅ-galactosamine derivative **2** with a ᴅ-galactose thioglycoside derivative **3** in the presence of a combination [26–27] of *N*-iodosuccinimide (NIS) and perchloric acid supported over silica (HClO_4_/SiO_2_) [28–29] furnished disaccharide derivative **8** in 79% yield, which on de-O-acetylation using sodium methoxide [30] gave the disaccharide acceptor **9** in 95% yield. NMR spectral analysis of compound **9** confirmed its formation with appropriate configuration at the glycosidic linkages [Signals at δ 5.54 (d, *J* = 2.5 Hz, H-1_A_), 5.44 (s, PhC*H*), 4.54 (d, *J* = 7.5 Hz, H-1_B_) in ^1^H NMR and at δ 105.2 (C-1_B_), 100.6 (Ph*C*H), 98.2 (C-1_A_) in ^13^C NMR spectra] (Scheme 1).

**Scheme 2:**
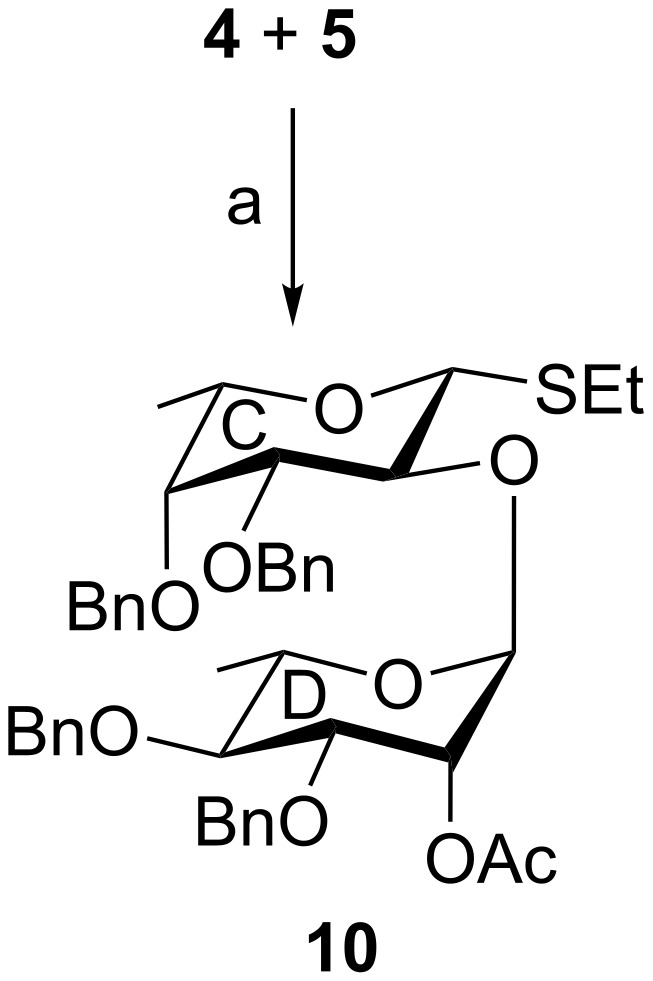
(a) HClO_4_/SiO_2_, CH_2_Cl_2_, −10 °C, 1 h, 76%.

In another experiment, ʟ-rhamnosyl trichloroacetimidate donor **5** was coupled with ʟ-fucosyl thioglycoside acceptor **4** in the presence of HClO_4_/SiO_2_ [31] as activator using an orthogonal glycosylation approach to furnish disaccharide thioglycoside derivative **10** in 76% yield, which was directly used in the next level of glycosylation. NMR spectral analysis of compound **10** unambiguously confirmed its formation [signals at δ 5.26 (d, *J* = 1.5 Hz, H-1_D_), 4.23 (d, *J* = 9.5 Hz, H-1_C_) in ^1^H NMR and at δ 98.4 (C-1_D_), 84.8 (C-1_C_) in ^13^C NMR spectra] (Scheme 2). It is worth noting that sulfide linkage at the anomeric position of compound **4** remained unaffected under the reaction conditions.

**Scheme 3:**
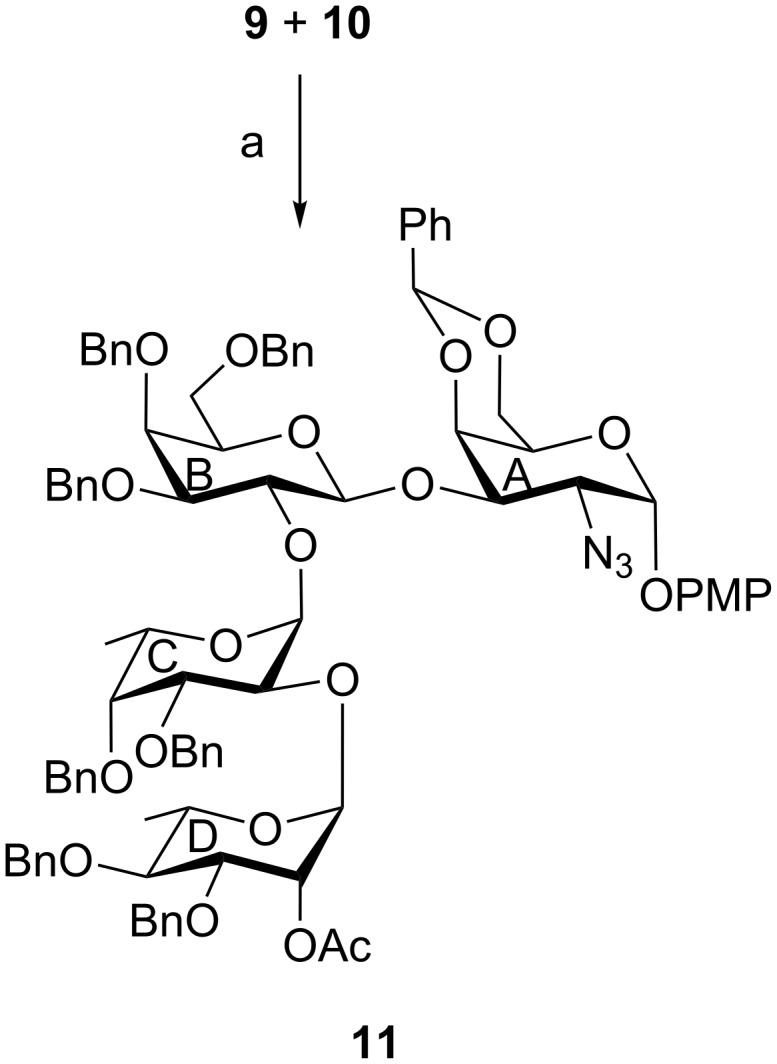
(a) NIS, HClO_4_/SiO_2_, MS 4 Å, CH_2_Cl_2_, −40 °C, 1 h, 22%.

**Scheme 4:**
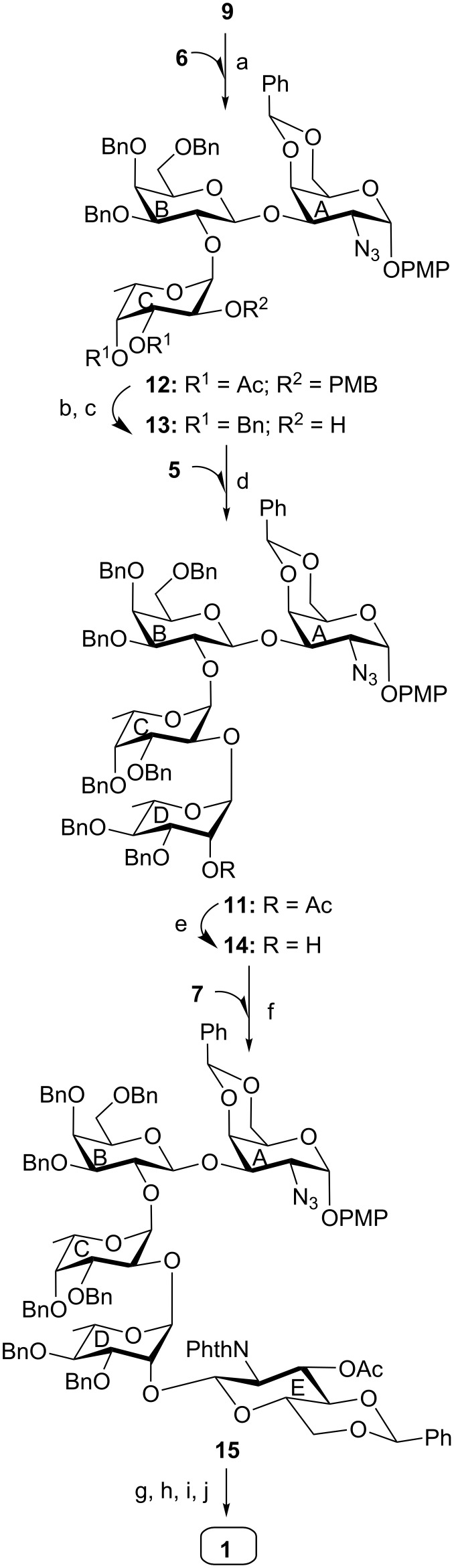
(a) NIS, HClO_4_/SiO_2_, MS 4 Å, CH_2_Cl_2_, −45 °C, 1 h, 74%; (b) BnBr, NaOH, TBAB, THF, room temperature, 6 h; (c) DDQ, CH_2_Cl_2_/H_2_O (9:1), room temperature, 2 h, 72% in two steps; (d) HClO_4_/SiO_2_, CH_2_Cl_2_, −10 °C, 1 h, 76%; (e) 0.1 M CH_3_ONa, CH_3_OH, room temperature, 2 h, 94%; (f) NIS, HClO_4_/SiO_2_, MS 4 Å, CH_2_Cl_2_, –15 °C, 1 h, 70%; (g) CH_3_COSH, pyridine, room temperature, 16 h; (h) NH_2_NH_2_·H_2_O, EtOH, 80 °C, 12 h; (i) Ac_2_O, CH_3_OH, room temperature, 30 min; (j) H_2_, 20%-Pd(OH)_2_/C, CH_3_OH, room temperature, 24 h, 49% in four steps.

Having achieved the disaccharide acceptor **9** and the disaccharide thioglycoside donor **10**, a stereoselective glycosylation between them was attempted in the presence of a combination [26–27] of NIS and HClO_4_/SiO_2_ as thiophilic activator. Unfortunately, the required tetrasaccharide derivative **11** was obtained in a poor yield (22%, Scheme 3). It was decided to follow a sequential glycosylation strategy to achieve a significant quantity of compound **11**. Accordingly, a stereoselective glycosylation was carried out using compound **9** with ʟ-fucose thioglycoside derivative **6** in the presence of a combination [26–27] of NIS and HClO_4_/SiO_2_ as thiophilic activator. Gratifyingly, the trisaccharide derivative **12** was obtained in 74% yield with a newly formed 1,2-*cis* glycosyl linkage in it. The structural confirmation of compound **12** was established by its NMR spectral analysis [signals at δ 5.67 (d, *J* = 3.0 Hz, H-1_A_), 5.60 (d, *J* = 3.5 Hz, H-1_C_), 5.50 (s, PhC*H*), 4.79 (d, *J* = 7.5 Hz, H-1_B_) in ^1^H NMR and at δ 103.3 (C-1_B_), 100.8 (Ph*C*H), 99.0 (C-1_A_), 97.1 (C-1_C_) in ^13^C NMR spectra]. Compound **12** was subjected to a set of reactions consisting of a one-pot [32] de-O-acetylation and benzylation using benzyl bromide and sodium hydroxide in the presence of tetrabutylammonium bromide (TBAB) followed by oxidative removal [33] of the PMB group using 2,3-dichloro-5,6-dicyano-1,4-benzoquinone (DDQ) to give trisaccharide acceptor **13** in 72% yield. Trisaccharide acceptor **13** was then allowed to couple with ʟ-rhamnosyl trichloroacetimidate donor **5** in the presence of HClO_4_/SiO_2_ as a solid acid activator [31] to provide tetrasaccharide derivative **11** in 76% yield, which was de-O-acetylated to furnish tetrasaccharide acceptor **14** in 94% yield. The formation of compound **11** with appropriate configuration at the glycosidic linkages was supported by its NMR spectral analysis [signals at δ 5.72 (d, *J* = 3.5 Hz, H-1_A_), 5.58 (d, *J* = 3.5 Hz, H-1_C_), 5.51 (s, PhC*H*), 4.80 (d, *J* = 7.5 Hz, H-1_B_), 4.71 (br s, H-1_D_) in ^1^H NMR and at δ 103.3 (C-1_B_), 100.6 (Ph*C*H), 99.0 (C-1_C_), 95.0 (C-1_A_), 94.1 (C-1_D_) in ^13^C NMR spectra]. Finally, NIS and HClO_4_/SiO_2_-promoted stereoselective glycosylation of compound **14** with ᴅ-glucosamine thioglycoside donor **7** furnished the desired pentasaccharide derivative **15** in 70% yield. The formation of compound **15** with appropriate configuration at the glycosidic linkages was supported by its NMR spectral analysis [signals at δ 5.64 (d, *J* = 3.5 Hz, H-1_A_), 5.54 (d, *J* = 3.0 Hz, H-1_C_), 5.50 (d, *J* = 8.5 Hz, H-1_E_), 5.48 (s, PhC*H*), 5.41 (s, PhC*H*), 4.96 (br s, H-1_D_), 4.69 (d, *J* = 8.0 Hz, H-1_B_) in ^1^H NMR and at δ 103.4 (C-1_B_), 101.6, 100.4 (2 C, 2 Ph*C*H), 100.1 (C-1_E_), 99.0 (C-1_C_), 94.9 (C-1_D_), 94.7 (C-1_A_) in ^13^C NMR spectra]. Compound **15** was subjected to a sequence of reactions consisting of (i) reductive transformation of the azido group into an acetamido group by the treatment with thioacetic acid [34]; (ii) transformation of the *N*-phthalimido group into acetamido group using hydrazine hydrate followed by selective N-acetylation [35]; (iii) hydrogenolysis of benzyl ethers and benzylidene acetals over Pearlman’s catalyst [36] to furnish the desired pentasaccharide **1** in 49% overall yield (Scheme 4). The structure of compound **1** was unambiguously characterized by its NMR spectral analysis [signals at δ 5.37 (d, *J* = 2.0 Hz, H-1_A_), 5.29 (d, *J* = 3.5 Hz, H-1_C_), 5.12 (br s, H-1_D_), 4.73 (d, *J* = 7.5 Hz, H-1_E_), 4.61 (d, *J* = 8.0 Hz, H-1_B_) in ^1^H NMR and at δ 102.2 (C-1_E_), 102.1 (C-1_B_), 96.8 (C-1_A_), 96.5 (C-1_C_), 96.0 (C-1_D_) in ^13^C NMR spectra].

## Conclusion

In summary, a convenient stepwise synthetic strategy has been developed for the synthesis of the pentasaccharide repeating unit of the cell wall O-antigen of *Escherichia albertii* O4 in very good yield. Although the target compound can be achieved by block synthetic approach but a better yield of the product was obtained by a sequential approach. HClO_4_/SiO_2_ was used as a solid acid activator in the glycosylation reactions using trichloroacetimidate as well as thioglycoside donors. All intermediate steps were high yielding with excellent stereo outcome in the glycosidic linkages.

## Supporting Information

File 1 Experimental and analytical data and copies of NMR spectra.
