# Supplementary material for: Convenient synthesis of the pentasaccharide repeating unit corresponding to the cell wall O-antigen of Escherichia albertii O4
Source: Beilstein J Org Chem. 2020 Jan 22;16:106–10. doi: 10.3762/bjoc.16.12 (PMC7006483; doi:10.3762/bjoc.16.12)
Supplement: File 1 — Experimental and analytical data and copies of NMR spectra. [file Beilstein_J_Org_Chem-16-106-s001.pdf]

BEILSTEIN JOURNAL OF ORGANIC CHEMISTRY

Supporting Information

for

Convenient synthesis of the pentasaccharide repeating unit corresponding to the cell wall O-antigen of *Escherichia albertii* O4

Tapasi Manna, Arin Gucchait and Anup Kumar Misra

Beilstein J. Org. Chem. **2020,** *16,* 106–110. doi:10.3762/bjoc.16.12

Experimental and analytical data and copies of NMR spectra

License and Terms: This is a supporting information file under the terms of the Creative Commons Attribution License (https://creativecommons.org/licenses/by/4.0). Please note that the reuse, redistribution and reproduction in particular requires that the authors and source are credited.
The license is subject to the *Beilstein Journal of Organic Chemistry* terms and conditions: (https://www.beilstein-journals.org/bjoc)

Index

Subject	Page No.
Detailed experimental methods for the synthesis of compounds **1**, **8–15**	**S2–S9**
1D and 2D NMR spectra of compounds **1, 8–15**	**S10–S27**

Experimental

General methods: All reactions were monitored by thin layer chromatography over silica gel coated TLC plates. The spots on TLC were visualized by warming ceric sulphate (2% $Ce(SO_4)_2$ in 2N H_2SO_4) sprayed plates in hot plate. Silica gel 230-400 mesh was used for column chromatography. NMR spectra were recorded on Bruker Avance 500 MHz using $CDCl_3$ as solvent and TMS as internal reference unless stated otherwise. Chemical shift value is expressed in δ ppm. The complete assignment of proton and carbon spectra was carried out by using a standard set of NMR experiments, e.g. ^{1}H NMR, ^{13}C NMR, ^{13}C DEPT 135, 2D COSY and 2D HSQC etc. HRMS were recorded on a Bruker mass spectrometer. Optical rotations were recorded in a Jasco P-2000 spectrometer. Commercially available grades of organic solvents of adequate purity are used in all reactions.

Preparation of $HClO_4/SiO_2$ catalyst: $HClO_4$ (3.6 g, 25 mmol, as a 70% aq solution) was added to a suspension of SiO_2 (230–400 mesh, 47.5 g) in Et_2O (140 mL). The mixture was concentrated and the residue was heated at 100 °C for 72 h under vacuum to furnish $HClO_4/SiO_2$ (0.5 mmol/g) as a free flowing powder. **Caution!:** Although no explosions were reported under these conditions, special care should be taken for large-scale preparation.

***p*-Methoxyphenyl (2-*O*-acetyl-3,4,6-tri-*O*-benzyl-β-D-galactopyranosyl)-(1→3)-2-azido-4,6-*O*-benzylidene-2-deoxy-α-D-galactopyranoside (8):** To a solution of compound **2** (1.5 g, 3.76 mmol) and compound **3** (2.1 g, 3.91 mmol) in CH_2Cl_2 (20 mL) was added MS 4 Å (2 g) and the reaction mixture was cooled to −45 °C under argon. To the cooled reaction mixture was added NIS (900 mg, 4.0 mmol) followed by $HClO_4/SiO_2$ (40 mg) and it was allowed to stir at the same temperature for 1 h. The reaction mixture was filtered and washed with CH_2Cl_2 (50 mL). The organic layer was successively washed with 5% aq. $Na_2S_2O_3$ (50 mL), H_2O (50 mL), dried (Na_2SO_4) and concentrated. The crude product was purified over SiO_2 using hexane-EtOAc (4:1) as eluant to give pure compound **8** (2.6 g, 79%). Colorless oil; $[\alpha]_D$ −7 (*c* 1.0, $CHCl_3$); ^{1}H NMR (500 MHz, $CDCl_3$): δ 7.30-7.21 (m, 20 H, Ar-H), 7.00 (d, *J* = 9.0 Hz, 2 H, Ar-H), 6.79 (d, *J* = 9.0 Hz, 2 H, Ar-H), 5.52 (d, *J* = 2.5 Hz, 1 H, H-1_A), 5.46 (s, 1 H, PhC*H*), 5.45 (t, *J* = 8.5 Hz, 1 H, H-2_B), 4.96 (d, *J* = 11.5 Hz, 1 H, PhC*H*), 4.72 (d, *J* = 8.0 Hz, 1 H, H-1_B), 4.67 (d, *J* = 12.0 Hz, 1 H, PhC*H*), 4.58-4.52 (m, 2 H, 2 PhC*H*), 4.40-4.39 (m, 3 H, H-4_A, 2 PhC*H*), 4.29 (dd, *J* = 9.0 Hz,

3.0 Hz, 1 H, H-3$_A$), 4.14 (d, *J* = 12.5 Hz, 1 H, H-6$_{aA}$), 3.91 (br s, 1 H, H-4$_B$), 3.89 (dd, *J* = 9.0 Hz, 3.0 Hz, 1 H, H-2$_A$), 3.82 (d, *J* = 12.5 Hz, 1 H, H-6$_{bA}$), 3.74 (s, 3 H, OC*H*$_3$), 3.68 (br s, 1 H, H-5$_B$), 3.65-3.64 (m, 2 H, H-6$_{abB}$), 3.60-3.58 (m, 1 H, H-5$_A$), 3.55 (dd, *J* = 9.0 Hz, 3.0 Hz, 1 H, H-3$_B$), 2.02 (s, 3 H, COC*H*$_3$); ^{13}C NMR (125 MHz, $CDCl_3$): δ 169.3 (*C*OCH$_3$), 155.2-114.6 (Ar-C), 102.4 (C-1$_B$), 100.3 (Ph*C*H), 98.4 (C-1$_A$), 80.7 (C-3$_B$), 75.5 (C-4$_A$), 74.4 (C-3$_A$), 74.3 (Ph*C*H$_2$), 73.9 (C-4$_B$), 73.5 (Ph*C*H$_2$), 72.7 (C-5$_A$), 72.2 (Ph*C*H$_2$), 71.1 (C-2$_B$), 69.2 (C-6$_B$), 68.9 (C-6$_A$), 63.8 (C-5$_B$), 58.7 (C-2$_A$), 55.5 (O*C*H$_3$), 20.9 (CO*C*H$_3$); HRMS $[M+Na]^+$: Calcd. 896.3371; found, 896.3380.

***p*-Methoxyphenyl (3,4,6-tri-*O*-benzyl-β-D-galactopyranosyl)-(1→3)-2-azido-4,6-*O*-benzylidene-2-deoxy-α-D-galactopyranoside (9)**: A solution of compound **8** (2 g, 2.29 mmol) in 0.1 M CH_3ONa in CH_3OH (25 mL) was allowed to stir at room temperature for 2 h and neutralized with Dowex 50W X8 (H^+) resin. The reaction mixture was filtered and the filtrate was concentrated under reduced pressure to give pure compound **9** (1.8 g, 95%). Colorless oil; $[\alpha]_D$ −7 (*c* 1.0, $CHCl_3$); ^{1}H NMR (500 MHz, $CDCl_3$): δ 7.27-7.22 (m, 20 H, Ar-H), 7.02 (d, *J* = 9.0 Hz, 2 H, Ar-H), 6.80 (d, *J* = 9.0 Hz, 2 H, Ar-H), 5.54 (d, *J* = 2.5 Hz, 1 H, H-1$_A$), 5.44 (s, 1 H, PhC*H*), 4.94 (d, *J* = 11.5 Hz, 1 H, PhC*H*), 4.85 (d, *J* = 12.0 Hz, 1 H, PhC*H*), 4.74 (d, *J* = 12.0 Hz, 1 H, PhC*H*), 4.59 (d, *J* = 11.5 Hz, 1 H, PhC*H*), 4.54 (d, *J* = 7.5 Hz, 1 H, H-1$_B$), 4.41-4.39 (m, 3 H, H-4$_A$, 2 PhC*H*), 4.32 (dd, *J* = 10.5 Hz, 3.0 Hz, 1 H, H-3$_A$), 4.16 (d, *J* = 12.5 Hz, 1 H, H-6$_{aA}$), 4.06 (t, *J* = 9.5 Hz, 1 H, H-2$_B$), 4.00 (dd, *J* = 10.5 Hz, 3.0 Hz, 1 H, H-2$_A$), 3.86-3.82 (m, 2 H, H-5$_B$, H-6$_{bA}$), 3.74 (s, 3 H, OC*H*$_3$), 3.69 (br s, 1 H, H-4$_B$), 3.63-3.62 (m, 2 H, H-6$_{abB}$), 3.55-3.54 (m, 1 H, H-5$_A$), 3.49 (dd, *J* = 9.5 Hz, 2.5 Hz, 1 H, H-3$_B$); ^{13}C NMR (125 MHz, $CDCl_3$): δ 155.3-114.6 (Ar-C), 105.2 (C-1$_B$), 100.6 (Ph*C*H), 98.2 (C-1$_A$), 81.7 (C-3$_B$), 75.8 (C-4$_A$), 75.4 (C-3$_A$), 74.5 (Ph*C*H$_2$), 74.0 (C-4$_B$), 73.9 (C-5$_A$), 73.5 (Ph*C*H$_2$), 73.1 (Ph*C*H$_2$), 71.6 (C-2$_B$), 69.3 (C-6$_B$), 68.9 (C-6$_A$), 63.7 (C-5$_B$), 58.9 (C-2$_A$), 53.2 (O*C*H$_3$); HRMS $[M+Na]^+$: Calcd. 854.3265; found, 854.3272.

Ethyl (2-*O*-acetyl-3,4-di-*O*-benzyl-α-L-rhamnopyranosyl)-(1→2)-3,4-di-*O*-benzyl-1-thio-β-L-fucopyranoside (10): A solution of compound **4** (1.5 g, 3.86 mmol) and compound **5** (2.4 g, 4.52 mmol) in CH_2Cl_2 (20 mL) was cooled to −10 °C. To the cooled reaction mixture was added $HClO_4/SiO_2$ (50 mg) and it was allowed to stir at the same temperature for 1 h. The reaction mixture was diluted with satd. aq. $NaHCO_3$ (50 mL) and extracted with CH_2Cl_2 (50 mL). The

organic layer was washed with H_2O (50 mL), dried (Na_2SO_4) and concentrated. The crude product was purified over SiO_2 using hexane-EtOAc (6:1) as eluant to give pure compound **10** (2.2 g, 76%); Colorless oil; $[\alpha]_D$ + 28 (*c* 1.0, $CHCl_3$); ^{1}H NMR (500 MHz, $CDCl_3$): δ 7.32-7.20 (m, 20 H, Ar-H), 5.52-5.51 (m, 1 H, H-2_D), 5.26 (d, *J* = 1.5 Hz, 1 H, H-1_D), 4.91-4.87 (m, 2 H, 2 PhC*H*), 4.71 (d, *J* = 11.5 Hz, 1 H, PhC*H*), 4.62-4.54 (m, 4 H, 4 PhC*H*), 4.49 (d, *J* = 11.0 Hz, 1 H, PhC*H*), 4.23 (d, *J* = 9.5 Hz, 1 H, H-1_C), 4.12-4.06 (m, 1 H, H-5_D), 4.00 (t, *J* = 9.5 Hz, 1 H, H-2_C), 3.89 (dd, *J* = 9.5 Hz, 3.5 Hz, 1 H, H-3_D), 3.58 (d, *J* = 2.0 Hz, 1 H, H-4_C), 3.47-3.46 (m, 1 H, H-5_C), 3.40 (dd, *J* = 9.5 Hz, 3.0 Hz, 1 H, H-3_C), 3.38 (t, *J* = 9.5 Hz, 1 H, H-4_D), 2.79-2.62 (m, 2 H, SCH_2CH_3), 2.13 (s, 3 H, $COCH_3$), 1.29 (t, *J* = 4.5 Hz, 3 H, SCH_2CH_3), 1.20 (d, *J* = 6.5 Hz, 3 H, CCH_3), 1.09 (d, *J* = 6.0 Hz, 3 H, CCH_3); ^{13}C NMR (125 MHz, $CDCl_3$): δ 169.8 ($COCH_3$), 138.8-127.4 (Ar-C), 98.4 (C-1_D), 84.8 (C-1_C), 83.4 (C-3_C), 80.1 (C-4_D), 77.9 (C-3_D), 76.0 (C-4_C), 75.2 ($PhCH_2$), 74.5 (2 C, C-5_C, $PhCH_2$), 73.8 (C-2_C), 72.6 ($PhCH_2$), 71.4 ($PhCH_2$), 68.5 (C-2_D), 68.2 (C-5_D), 24.1 (SCH_2CH_3), 21.1 ($COCH_3$), 17.7 (CCH_3), 17.3 (CCH_3), 14.9 (SCH_2CH_3); HRMS $[M+Na]^+$: Calcd. 779.3230; found, 779.3238.

***p*-Methoxyphenyl (2-*O*-acetyl-3,4-di-*O*-benzyl-α-L-rhamnopyranosyl)-(1→2)-(3,4-di-*O*-benzyl-α-L-fucopyranosyl)-(1→2)-(3,4,6-tri-*O*-benzyl-β-D-galactopyranosyl)-(1→3)-2-azido-4,6-*O*-benzylidene-2-deoxy-α-D-galactopyranoside (11)**: To a solution of compound **9** (0.4 g, 0.48 mmol) and compound **10** (0.4 g, 0.53 mmol) in CH_2Cl_2 (10 mL) was added MS 4Å (1 g) and the reaction mixture was cooled to −40 °C under argon. To the cooled reaction mixture was added NIS (130 mg, 0.58 mmol) followed by $HClO_4/SiO_2$ (10 mg) and it was allowed to stir at the same temperature for 1 h. The reaction mixture was filtered and washed with CH_2Cl_2 (20 mL). The organic layer was successively washed with 5% aq. $Na_2S_2O_3$ (20 mL), H_2O (20 mL), dried (Na_2SO_4) and concentrated. The crude product was purified over SiO_2 using hexane-EtOAc (4:1) as eluant to give pure compound **11** (160 mg, 22%).

Following sequential strategy: A solution of compound **13** (700 mg, 0.60 mmol) and compound **5** (380 mg, 0.72 mmol) in CH_2Cl_2 (5 mL) was cooled to −10 °C under argon. To the cooled reaction mixture was added $HClO_4/SiO_2$ (10 mg) and it was allowed to stir at same temperature for 1 h. The reaction mixture was filtered and washed with CH_2Cl_2 (50 mL). The organic layer was successively washed with 5% aq. $Na_2S_2O_3$ (50 mL), H_2O (50 mL), dried (Na_2SO_4) and concentrated. The crude product was purified over SiO_2 using hexane-EtOAc (4:1) as eluant to

give pure compound **11** (700 mg, 76%). Colorless oil; $[\alpha]_D$ – 17 (*c* 1.0, $CHCl_3$); ^{1}H NMR (500 MHz, $CDCl_3$): δ 7.46-6.81 (m, 44 H, Ar-H), 5.72 (d, *J* = 3.5 Hz, 1 H, H-1_A), 5.58 (d, *J* = 3.5 Hz, 1 H, H-1_C), 5.51 (s, 1 H, PhC*H*), 5.44-5.43 (m, 1 H, H-2_D), 4.83 (d, *J* = 11.0 Hz, 1 H, PhC*H*), 4.82 (d, *J* = 11.0 Hz, 1 H, PhC*H*), 4.80 (d, *J* = 7.5 Hz, 1 H, H-1_B), 4.73 (d, *J* = 11.5 Hz, 1 H, PhC*H*), 4.71 (br s, 1 H, H-1_D), 4.69 (d, *J* = 11.5 Hz, 1 H, PhC*H*), 4.66 (d, *J* = 11.0 Hz, 1 H, PhC*H*), 4.63 (d, *J* = 11.0 Hz, 1 H, PhC*H*), 4.56 (d, *J* = 11.0 Hz, 1 H, PhC*H*), 4.54 (d, *J* = 11.0 Hz, 1 H, PhC*H*), 4.53 (d, *J* = 11.0 Hz, 1 H, PhC*H*), 4.50 (d, *J* = 11.0 Hz, 1 H, PhC*H*), 4.48 (d, *J* = 11.5 Hz, 1 H, PhC*H*), 4.45-4.36 (m, 5 H, H-3_A, H-4_A, 3 PhC*H*), 4.30 (dd, *J* = 9.0 Hz, 3.0 Hz, 1 H, H-3_C), 4.26 (t, *J* = 8.0 Hz, 1 H, H-2_B), 4.18-4.16 (m, 2 H, H-5_B, H-6_{aA}), 4.15-4.12 (m, 1 H, H-5_D), 3.99 (br s, 1 H, H-4_C), 3.97 (dd, *J* = 8.0 Hz, 3.0 Hz, 1 H, H-3_D), 3.92-3.89 (m, 2 H, H-3_B, H-6_{bA}), 3.77 (s, 3 H, OCH_3), 3.77-3.72 (m, 3 H, H-2_C, H-5_A, H-5_C,), 3.65-3.62 (m, 2 H, H-6_{abB}), 3.60 (dd, *J* = 9.0 Hz, 3.0 Hz, 1 H, H-2_A), 3.48 (br s, 1 H, H-4_B), 3.26 (t, *J* = 8.0 Hz, 1 H, H-4_D), 1.84 (s, 3 H, COCH_3), 1.16 (d, *J* = 6.0 Hz, 3 H, CCH_3), 0.91 (d, *J* = 6.0 Hz, 3 H, CCH_3); ^{13}C NMR (125 MHz, $CDCl_3$): δ 170.0 (*C*OCH_3), 155.4-114.7 (Ar-C), 103.3 (C-1_B), 100.6 (Ph*C*H), 99.0(C-1_C), 95.0 (C-1_A), 94.1 (C-1_D), 84.3 (C-3_C) 80.0 (C-4_D), 78.3 (C-4_B), 78.1 (C-3_D), 77.6 (C-4_A), 76.1 (C-5_C), 74.9, 74.8, 74.2 (3 C, 3 Ph*C*H_2), 73.5 (C-3_B), 73.4 (Ph*C*H_2), 73.1 (C-5_A), 72.9, 716 (2 C, 2 Ph*C*H_2), 71.3 (C-5_B), 70.7 (C-4_C), 70.5 (Ph*C*H_2), 70.3 (C-2_B), 69.0 (C-6_A), 68.9 (C-2_D), 68.8 (C-6_B), 67.3 (C-5_D), 66.2 (C-3_A), 63.9 (C-2_C), 58.7 (C-2_A), 55.5 (O*C*H_3), 20.9 (CO*C*H_3), 17.6 (C*C*H_3), 16.2 (C*C*H_3); HRMS $[M+Na]^+$: Calcd. 1548.6407; found, 1548.6400.

***p*-Methoxyphenyl (3,4-di-*O*-acetyl-2-*O*-(*p*-methoxybenzyl))-α-L-fucopyranosyl)-(1→2)-(3,4,6-tri-*O*-benzyl-β-D-galactopyranosyl)-(1→3)-2-azido-4,6-*O*-benzylidene-2-deoxy-α-D-galactopyranoside (12)**: To a solution of compound **9** (1.2 g, 1.44 mmol) and compound **6** (625 mg, 1.51 mmol) in CH_2Cl_2 (10 mL) was added MS 4 Å (1 g) and the reaction mixture was cooled to −45 °C under argon. To the cooled reaction mixture was added NIS (350 mg, 1.56 mmol) followed by $HClO_4/SiO_2$ (20 mg) and it was allowed to stir at the same temperature for 1 h. The reaction mixture was filtered and washed with CH_2Cl_2 (50 mL). The organic layer was successively washed with 5% aq. $Na_2S_2O_3$ (50 mL), H_2O (50 mL), dried (Na_2SO_4) and concentrated. The crude product was purified over SiO_2 using hexane-EtOAc (4:1) as eluant to give pure compound **12** (1.26 g, 74%). Colorless oil; $[\alpha]_D$ + 10 (*c* 1.0, $CHCl_3$); ^{1}H NMR (500 MHz, $CDCl_3$): δ 7.47-6.63 (m, 28 H, Ar-H), 5.67 (d, *J* = 3.0 Hz, 1 H, H-1_A), 5.60 (d, *J* = 3.5 Hz,

1 H, H-1$_C$), 5.50 (s, 1 H, PhC*H*), 5.44 (dd, *J* = 10.5 Hz, 3.0 Hz, 1 H, H-3$_C$), 5.12 (br s, 1 H, H-4$_C$), 4.85 (d, *J* = 11.5 Hz, 1 H, PhC*H*), 4.79 (d, *J* = 7.5 Hz, 1 H, H-1$_B$), 4.73-4.70 (m, 2 H, H-5$_A$, PhC*H*), 4.60 (d, *J* = 12.0 Hz, 1 H, PhC*H*), 4.52 (d, *J* = 11.5 Hz, 1 H, PhC*H*), 4.43 (br s, 2 H, PhC*H*), 4.41-4.34 (m, 3 H, H-4$_A$, H-5$_B$, PhC*H*), 4.28 (d, *J* = 12.0 Hz, 1 H, PhC*H*), 4.22-4.16 (m, 2 H, H-2$_B$, H-6$_{aA}$), 4.06-4.05 (m, 2 H, H-6$_{abB}$), 3.92-3.90 (m, 2 H, H-4$_B$, H-6$_{bA}$), 3.81 (s, 3 H, OCH_3), 3.80-3.76 (m, 2 H, H-2$_A$, H-3$_A$), 3.72 (s, 3 H, OCH_3), 3.72-3.71 (m, 2 H, H-2$_C$, H-5$_C$), 3.65-3.63 (m, 1 H, H-3$_B$), 2.03 (s, 3 H, COCH_3), 1.93 (s, 3 H, COCH_3), 0.70 (d, *J* = 6.0 Hz, 3 H, CCH_3); ^{13}C NMR (125 MHz, CDCl$_3$): δ 170.0, 169.6 (2 C, 2 *C*OCH$_3$), 155.3-113.4 (Ar-C), 103.3 (C-1$_B$), 100.8 (Ph*C*H), 99.0 (C-1$_A$), 97.1 (C-1$_C$), 84.1 (C-2$_C$), 76.1 (C-4$_A$), 74.4 (Ph*C*H$_2$), 73.6 (2C, C-3$_B$, C-5$_B$), 73.5 (Ph*C*H$_2$), 72.5 (C-4$_C$), 72.4 (C-2$_B$), 72.3 (C-4$_B$), 72.0 (C-5$_C$), 71.6, 71.5 (2 C, 2 Ph*C*H$_2$), 70.0 (C-3$_C$), 68.8 (C-6$_A$), 66.8 (C-6$_B$), 64.3 (C-5$_A$), 63.8 (C-3$_A$), 58.2 (C-2$_A$), 55.4 (O*C*H$_3$), 55.0 (O*C*H$_3$), 23.0, 22.7 (2 C, 2 CO*C*H$_3$), 15.2 (C*C*H$_3$); HRMS [M+Na]$^+$: Calcd. 1204.4631; found, 1204.4640.

***p*-Methoxyphenyl (3,4-di-*O*-benzyl-α-L-fucopyranosyl)-(1→2)-(3,4,6-tri-*O*-benzyl-β-D-galactopyranosyl)-(1→3)-2-azido-4,6-*O*-benzylidene-2-deoxy-α-D-galactopyranoside (13)**: To a solution of compound **12** (1.1 g, 0.93 mmol) in THF (25 mL) were added benzyl bromide (550 μL, 4.63 mmol), powdered NaOH (750 mg, 18.75 mmol) and TBAB (50 mg) and the reaction mixture was vigorously stirred at room temperature for 6 h. The reaction mixture was diluted with H_2O (100 mL) and extracted with CH_2Cl_2 (100 mL). The organic layer was washed with H_2O (50 mL), dried (Na_2SO_4) and concentrated. To a solution of the crude product in CH_2Cl_2 (50 mL) was added DDQ (500 mg, 2.20 mmol) in H_2O (10 mL) and the reaction mixture was stirred at room temperature for 2 h. The reaction mixture was diluted with satd. $NaHCO_3$ (50 mL) and extracted with CH_2Cl_2 (50 mL). The organic layer was washed with H_2O (50 mL), dried (Na_2SO_4) and concentrated. The crude product was purified over SiO_2 using hexane-EtOAc (5:1) as eluant to give pure compound **13** (775 mg, 72%). Colorless oil; [α]$_D$ + 42 (*c* 1.0, CHCl$_3$); ^{1}H NMR (500 MHz, CDCl$_3$): δ 7.45-6.80 (m, 34 H, Ar-H), 5.58 (d, *J* = 3.0 Hz, 1 H, H-1$_A$), 5.50 (s, 1 H, PhC*H*), 5.35 (d, *J* = 4.0 Hz, 1 H, H-1$_C$), 4.89-4.85 (m, 2 H, H-5$_A$, PhC*H*), 4.67 (d, *J* = 11.0 Hz, 1 H, PhC*H*), 4.61 (d, *J* = 7.5 Hz, 1 H, H-1$_B$), 4.57-4.51 (m, 3 H, 3 PhC*H*), 4.44-4.38 (m, 6 H, H-4$_A$, 5 PhC*H*), 4.33 (dd, *J* = 11.0 Hz, 3.5 Hz, H-3$_C$), 4.24 (t, *J* = 8.0 Hz, 1 H, H-2$_B$), 4.18 (d, *J* = 12.0 Hz, 1 H, H-6$_{aA}$), 3.94-3.93 (m, 2 H, H-4$_B$, H-4$_C$), 3.92-3.91 (m, 2 H, H-5$_B$, H-6$_{bA}$), 3.76 (s, 3

H, OCH_3), 3.76-3.74 (m, 2 H, H-3$_A$, H-5$_C$), 3.72 (dd, *J* = 9.0 Hz, 3.0 Hz, 1 H, H-2$_A$), 3.65-3.64 (m, 2 H, H-2$_C$, H-6$_{aB}$), 3.63-3.57 (m, 2 H, H-3$_B$, H-6$_{bB}$), 0.95 (d, *J* = 6.0 Hz, 3 H, CCH_3); ^{13}C NMR (125 MHz, $CDCl_3$): δ 155.5-114.7 (Ar-C), 103.1 (C-1$_B$), 100.9 (Ph*C*H), 98.7 (C-1$_A$), 95.5 (C-1$_C$), 83.3 (C-3$_B$), 81.9 (C-5$_B$), 80.0 (C-5$_A$), 76.0 (C-4$_A$), 74.6 (2 C, 2 Ph*C*H_2), 73.8 (C-4$_B$), 73.5 (2 C, 2 Ph*C*H_2), 73.4 (C-4$_C$), 72.4 (C-3$_C$), 72.3 (C-2$_B$), 72.7 (Ph*C*H_2), 68.8 (C-6$_A$), 68.7 (C-6$_B$), 65.6 (C-2$_C$), 63.9 (C-3$_A$), 62.8 (C-5$_C$), 58.9 (C-2$_A$), 55.5 (O*C*H_3), 14.9 (C*C*H_3); HRMS [M+Na]$^+$: Calcd. 1180.4783; found, 1180.4790.

***p*-Methoxyphenyl (3,4-di-*O*-benzyl-α-L-rhamnopyranosyl)-(1→2)-(3,4-di-*O*-benzyl-α-L-fucopyranosyl)-(1→2)-(3,4,6-tri-*O*-benzyl-β-D-galactopyranosyl)-(1→3)-2-azido-4,6-*O*-benzylidene-2-deoxy-α-D-galactopyranoside (14)**: A solution of compound **11** (700 mg, 0.46 mmol) in 0.1 M CH_3ONa in CH_3OH (25 mL) was allowed to stir at room temperature for 2 h and neutralized with Dowex 50W X8 (H^+) resin. The reaction mixture was filtered and the filtrate was concentrated under reduced pressure to give pure compound **14** (640 mg, 94%). Colorless oil; $[\alpha]_D$ –97 (*c* 1.0, $CHCl_3$); ^{1}H NMR (500 MHz, $CDCl_3$): δ 7.47-6.80 (m, 44 H, Ar-H), 5.64 (d, *J* = 3.5 Hz, 1 H, H-1$_A$), 5.59 (d, *J* = 3.0 Hz, 1 H, H-1$_C$), 5.51 (s, 1 H, PhC*H*), 4.82 (br s, 1 H, H-1$_D$), 4.81 (d, *J* = 11.0 Hz, 1 H, PhC*H*), 4.80 (d, *J* = 11.0 Hz, 1 H, PhC*H*), 4.69 (d, *J* = 7.5 Hz, 1 H, H-1$_B$), 4.59-4.58 (m, 3 H, 3 PhC*H*), 4.51-4.39 (m, 7 H, H-4$_A$, 6 PhC*H*), 4.35 (dd, *J* = 9.0 Hz, 3.0 Hz, 1 H, H-3$_A$), 4.23 (t, *J* = 9.0 Hz, 1 H, H-2$_B$), 4.17 (d, *J* = 12.5 Hz, 1 H, H-6$_{aA}$), 3.96-3.94 (m, 2 H, H-3$_D$, H-5$_A$), 3.90 (d, *J* = 10.0 Hz, 1 H, H-6$_{bA}$), 3.87 (br s, 1 H, H-4$_C$), 3.83 (br s, 1 H, H-4$_B$), 3.82 (s, 3 H, OCH_3), 3.82-3.71 (m, 4 H, H-2$_A$, H-2$_D$, H-3$_B$, H-5$_B$), 3.70-3.62 (m, 3 H, H-2$_C$, H-5$_D$, H-6$_{aB}$), 3.58-3.57 (m, 2 H, H-3$_C$, H-6$_{bB}$), 3.50-3.49 (m, 1 H, H-5$_C$), 3.41 (t, *J* = 8.0 Hz, 1 H, H-4$_D$), 0.96 (d, *J* = 6.0 Hz, 3 H, CCH_3), 0.89 (d, *J* = 6.0 Hz, 3 H, CCH_3); ^{13}C NMR (125 MHz, $CDCl_3$): δ 155.3-114.7 (Ar-C), 103.4 (C-1$_B$), 100.7 (Ph*C*H), 99.2 (C-1$_D$), 99.0 (C-1$_C$), 96.6 (C-1$_A$), 84.6 (C-3$_C$), 79.7 (2 C, C-3$_B$, C-4$_D$), 78.4 (C-4$_B$), 76.0 (C-4$_A$), 75.3, 74.3 (2 C, 2 Ph*C*H_2), 73.6 (C-2$_B$), 73.4 (Ph*C*H_2), 73.2 (C-2$_C$), 72.2 (C-5$_D$), 72.1, 71.9 (3 C, 3 Ph*C*H_2), 71.8 (C-3$_A$), 71.7 (C-3$_D$), 69.0 (C-6$_A$), 68.9 (2 C, C-4$_C$, C-5$_A$), 68.8 (C-6$_B$), 68.4 (C-5$_B$), 65.3 (C-5$_C$), 65.1 (Ph*C*H_2), 63.9 (C-2$_D$), 58.8 (C-2$_A$), 55.5 (O*C*H_3), 15.6 (C*C*H_3), 14.1 (C*C*H_3); HRMS [M+Na]$^+$: Calcd. 1506.6301; found, 1506.6310.

***p*-Methoxyphenyl (2-*O*-acetyl-4,6-*O*-benzylidene-2-deoxy-2-*N*-phthalimido-β-D-glucopyranosyl)-(1→2)-(3,4-di-*O*-benzyl-α-L-rhamnopyranosyl)-(1→2)-(3,4-di-*O*-benzyl-α-**

L-fucopyranosyl)-(1→2)-(3,4,6-tri-*O*-benzyl-β-D-galactopyranosyl)-(1→3)-2-azido-4,6-*O*-benzylidene-2-deoxy-α-D-galactopyranoside (15): To a solution of compound **14** (600 mg, 0.40 mmol) and compound **7** (230 mg, 0.47 mmol) in CH_2Cl_2 (5 mL) was added MS 4 Å (0.5 g) and the reaction mixture was cooled to −15 °C under argon. To the cooled reaction mixture was added NIS (120 mg, 0.53 mmol) followed by $HClO_4$-SiO_2 (10 mg) and it was allowed to stir at the same temperature for 1 h. The reaction mixture was filtered and washed with CH_2Cl_2 (50 mL). The organic layer was successively washed with 5% aq. $Na_2S_2O_3$ (50 mL), H_2O (50 mL), dried (Na_2SO_4) and concentrated. The crude product was purified over SiO_2 using hexane-EtOAc (4:1) as eluant to give pure compound **15** (535 mg, 70%). Colorless oil; $[\alpha]_D$ – 50 (*c* 1.0, $CHCl_3$); ^{1}H NMR (500 MHz, $CDCl_3$): δ 7.57-6.82 (m, 53 H, Ar-H), 6.05 (t, *J* = 8.5 Hz, 1 H, H-3$_E$), 5.64 (d, *J* = 3.5 Hz, 1 H, H-1$_A$), 5.54 (d, *J* = 3.0 Hz, 1 H, H-1$_C$), 5.50 (d, *J* = 8.5 Hz, 1 H, H-1$_E$), 5.48 (s, 1 H, PhC*H*), 5.41 (s, 1 H, PhC*H*), 4.98 (d, *J* = 11.0 Hz, 1 H, PhC*H*), 4.96 (br s, 1 H, H-1$_D$), 4.76 (d, *J* = 11.5 Hz, 1 H, PhC*H*), 4.71 (d, *J* = 11.0 Hz, 1 H, PhC*H*), 4.69 (d, *J* = 8.0 Hz, 1 H, H-1$_B$), 4.63 (d, *J* = 11.0 Hz, 1 H, PhC*H*), 4.57 (d, *J* = 11.5 Hz, 1 H, PhC*H*), 4.51 (d, *J* = 11.0 Hz, 1 H, PhC*H*), 4.44-4.41 (m, 3 H, 3 PhC*H*), 4.38-4.27 (m, 7 H, H-2$_E$, H-4$_A$, 5 PhC*H*), 4.17-4.11 (m, 4 H, H-2$_B$, H-3$_A$, H-4$_E$, H-6$_{aA}$), 4.01 (dd, *J* = 9.0 Hz, 3.0 Hz, 1 H, H-3$_C$), 4.00 (br s, 1 H, H-4$_C$), 3.90 (d, *J* = 8.0 Hz, 1 H, H-6$_{bA}$), 3.84-3.79 (m, 3 H, H-2$_D$, H-3$_D$, H-4$_B$), 3.77 (s, 3 H, OC*H*$_3$), 3.77-3.74 (m, 2 H, H-2$_C$, H-3$_B$), 3.67-3.63 (m, 5 H, H-5$_A$, H-5$_B$, H-5$_D$, H-5$_E$, H-6$_{aB}$), 3.61-3.59 (m, 5 H, H-2$_A$, H-2$_C$, H-6$_{bB}$, H-6$_{abE}$), 3.36-3.32 (m, 1 H, H-5$_C$), 2.86 (t, *J* = 8.0 Hz, 1 H, H-4$_D$), 1.92 (s, 3 H, COC*H*$_3$), 1.02 (d, *J* = 6.0 Hz, 3 H, CC*H*$_3$), 0.84 (d, *J* = 6.0 Hz, 3 H, CC*H*$_3$); ^{13}C NMR (125 MHz, $CDCl_3$): δ 169.6 (*C*OCH$_3$), 155.3-114.7 (Ar-C), 103.4 (C-1$_B$), 101.6, 100.4 (2 C, 2 Ph*C*H), 100.1 (C-1$_E$), 99.0 (C-1$_C$), 94.9 (C-1$_D$), 94.7 (C-1$_A$), 84.1 (C-5$_E$), 80.9 (C-4$_D$), 79.4 (C-5$_D$), 78.7 (C-3$_D$), 78.0 (2C, C-3$_B$, C-3$_C$), 77.8 (C-5$_B$), 75.9 (C-5$_A$), 74.7, 74.5, 74.3 (3 C, 3 Ph*C*H$_2$), 73.6 (C-4$_B$), 73.4, 73.1 (2 C, 2 Ph*C*H$_2$), 72.9 (C-2$_D$), 72.5 (C-4$_C$), 72.4, 71.9 (2 C, 2 Ph*C*H$_2$), 71.1 (C-3$_A$), 70.4 (C-4$_E$), 69.2 (C-3$_E$), 68.8 (2 C, C-6$_A$, C-6$_B$), 68.7 (C-6$_E$), 68.0 (C-4$_A$), 66.2 (C-2$_B$), 65.6 (C-5$_C$), 63.8 (C-2$_C$), 58.7 (C-2$_A$), 55.5 (O*C*H$_3$), 55.3 (C-2$_E$), 20.6 (CO*C*H$_3$), 17.6 (C*C*H$_3$), 16.2 (C*C*H$_3$); HRMS $[M+Na]^+$: Calcd. 1927.7463; found, 1927.7470.

***p*-Methoxyphenyl (2-acetamido-2-deoxy-β-D-glucopyranosyl)-(1→2)-(α-L-rhamnopyranosyl)-(1→2)-(α-L-fucopyranosyl)-(1→2)-(β-D-galactopyranosyl)-(1→3)-2-acetamido-2-deoxy-α-D-galactopyranoside (1)**: To a solution of compound **15** (500 mg, 0.26

mmol) in pyridine (5 mL) was added CH_3COSH (0.5 mL, 7.1 mmol) and it was allowed to stir at room temperature for 16 h. The solvents were removed under reduced pressure and co-evaporated with toluene (3 × 10 mL). To a solution of the crude product in EtOH (15 mL) was added $NH_2NH_2{\cdot}H_2O$ (0.2 mL) and the reaction mixture was allowed to stir at 80 °C for 12 h. The solvents were removed under reduced pressure and a solution of the crude product in CH_3OH (5 mL) was treated with acetic anhydride (1 mL) at room temperature for 30 min. The solvents were removed under reduced pressure and co-evaporated with toluene (3 × 10 mL). To a solution of the crude product in CH_3OH (10 mL) was added 20% $Pd(OH)_2/C$ (100 mg) and the reaction mixture was allowed to stir at room temperature under a positive pressure of hydrogen for 24 h. The reaction mixture was filtered through a Celite bed and concentrated. The hydrogenolized product was passed through a Sephadex LH-20 column using CH_3OH-H_2O (5:1) as eluant to give pure compound **1** (125 mg, 49%). White powder; $[\alpha]_D$ (*c* 1.0, H_2O); ^1H NMR (500 MHz, $CDCl_3$): δ 7.00 (d, *J* = 9.0 Hz, 2 H, Ar-H), 6.89 (d, *J* = 9.0 Hz, 1 H, Ar-H), 5.37 (d, *J* = 2.0 Hz, 1 H, H-1_A), 5.29 (d, *J* = 3.5 Hz, 1 H, H-1_C), 5.12 (br s, 1 H, H-1_D), 4.73 (d, *J* = 7.5 Hz, 1 H, H-1_E), 4.61 (d, *J* = 8.0 Hz, 1 H, H-1_B), 4.26-4.22 (m, 2 H, H-2_A, H-5_C), 4.19-4.17 (m, 2 H, H-3_A, H-4_A), 4.11-4.09 (m, 1 H, H-5_A), 4.01-3.98 (m, 1 H, H-2_D), 3.86-3.79 (m, 6 H, H-2_C, H-3_B, H-3_D, H-4_C, H-5_D, H-6_{aA}), 3.71 (s, 3 H, OCH_3), 3.71-3.68 (m, 3 H, H-3_C, H-4_B, H-5_B), 3.66-3.60 (m, 7 H, H-2_B, H-2_E, H-6_{bA}, H-6_{abB}, H-6_{abE}), 3.49 (t, *J* = 9.0 Hz, 1 H, H-3_E), 3.41-3.39 (m, 1 H, H-5_E), 3.32 (t, *J* = 9.0 Hz, 1 H, H-4_E), 3.27 (t, *J* = 9.0 Hz, 1 H, H-4_D), 1.97 (s, 3 H, $NHCOCH_3$), 1.96 (s, 3 H, $NHCOCH_3$), 1.19 (d, *J* = 6.0 Hz, 3 H, CCH_3), 1.14 (d, *J* = 6.0 Hz, 3 H, CCH_3); ^{13}C NMR (125 MHz, $CDCl_3$): δ 174.7, 173.6 (2 C, 2 $NHCOCH_3$), 154.4-114.0 (Ar-C), 102.2 (C-1_E), 102.1 (C-1_B), 96.8 (C-1_A), 96.5 (C-1_C), 96.0 (C-1_D), 78.4 (C-2_D), 76.0 (C-5_E), 75.8 (C-2_B), 75.0 (C-3_B), 74.2 (C-3_E), 73.7 (C-5_C), 73.5 (C-5_B), 72.3 (C-5_A), 71.9 (C-2_C), 71.5 (C-4_C), 71.3 (C-4_B), 69.9 (C-4_E), 69.7 (C-3_D), 69.1 (C-5_D), 69.0 (C-3_A), 68.9 (C-4_D), 67.7 (C-3_C), 66.9 (C-4_A), 61.0 (C-6_E), 60.9 (C-6_B), 60.8 (C-6_A), 55.8 (C-2_E), 55.7 (OCH_3), 49.5 (C-2_A), 22.3 ($NHCOCH_3$), 21.8 ($NHCOCH_3$), 16.6 (CCH_3), 15.2 (CCH_3); HRMS $[M+Na]^+$: Calcd. 1007.3696; found, 1007.3704.

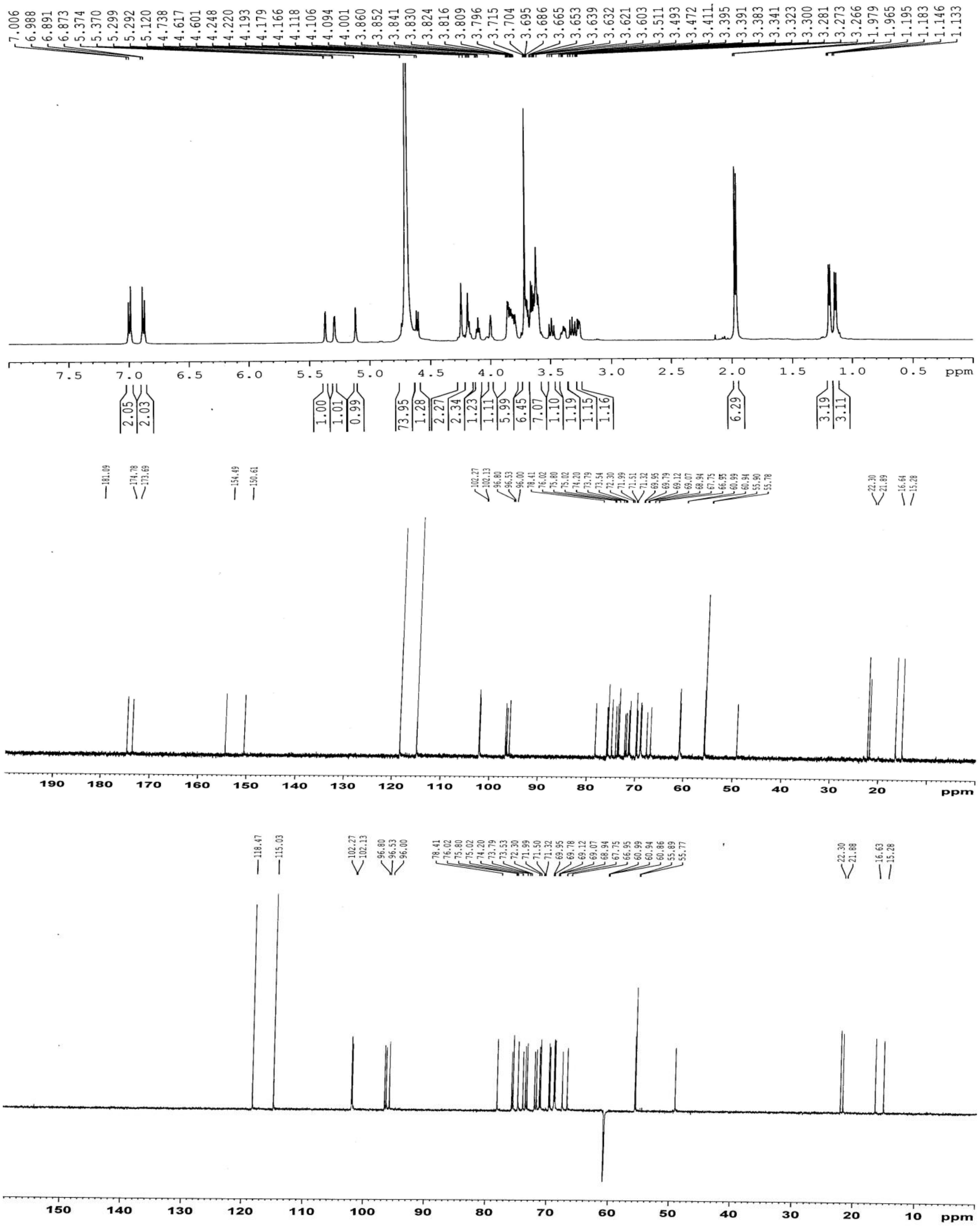

^{1}H, ^{13}C and DEPT 135 NMR spectra of *p*-methoxyphenyl (2-acetamido-2-deoxy-β-D-glucopyranosyl)-(1→2)-(α-L-rhamnopyranosyl)-(1→2)-(α-L-fucopyranosyl)-(1→2)-(β-D-galactopyranosyl)-(1→3)-2-acetamido-2-deoxy-α-D-galactopyranoside (**1**) (D_2O).

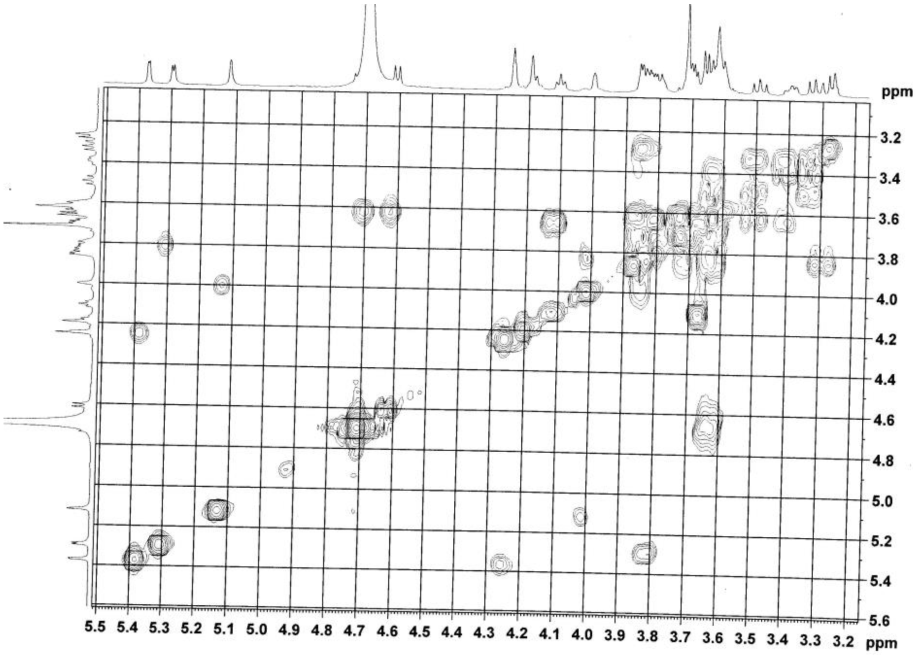

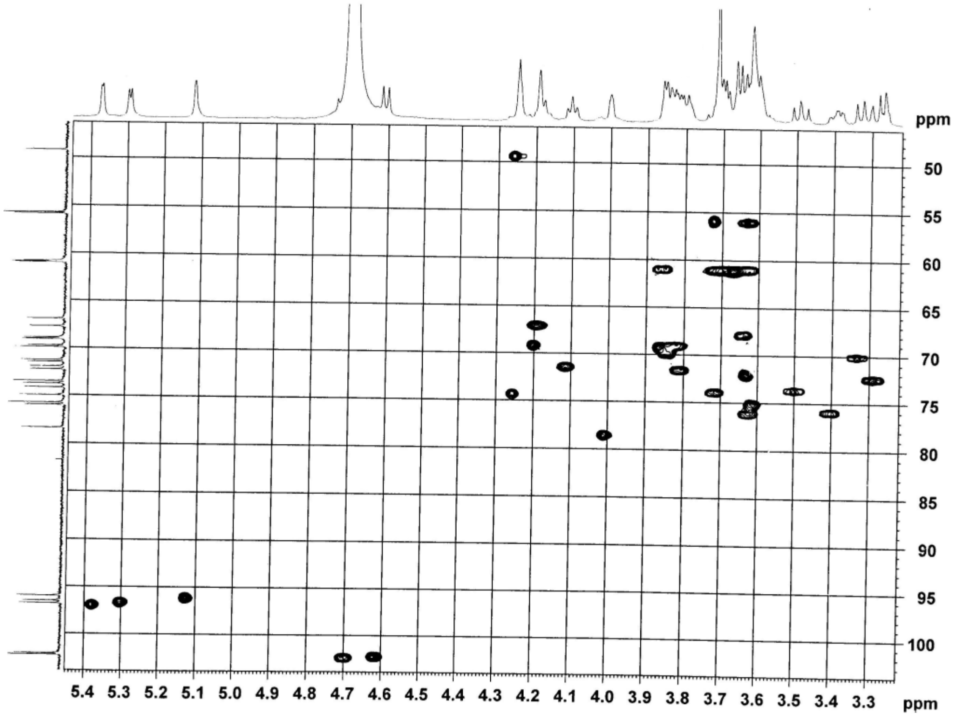

2D COSY and HSQC NMR spectra of *p*-methoxyphenyl (2-acetamido-2-deoxy-β-D-glucopyranosyl)-(1→2)-(α-L-rhamnopyranosyl)-(1→2)-(α-L-fucopyranosyl)-(1→2)-(β-D-galactopyranosyl)-(1→3)-2-acetamido-2-deoxy-α-D-galactopyranoside (**1**) (D_2O) (Selected region).

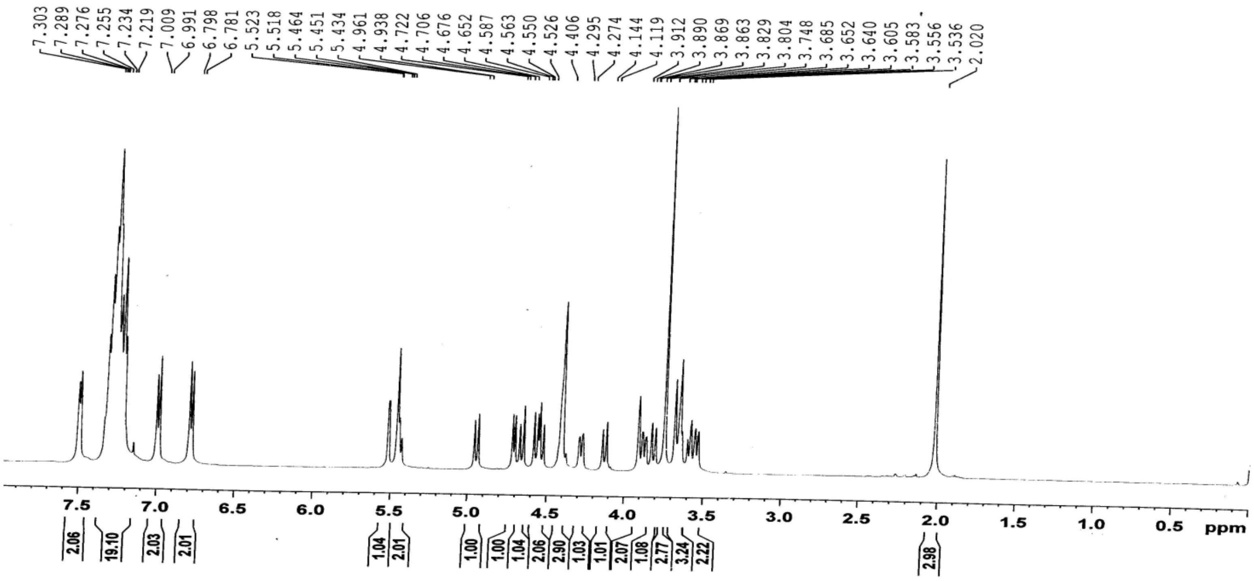

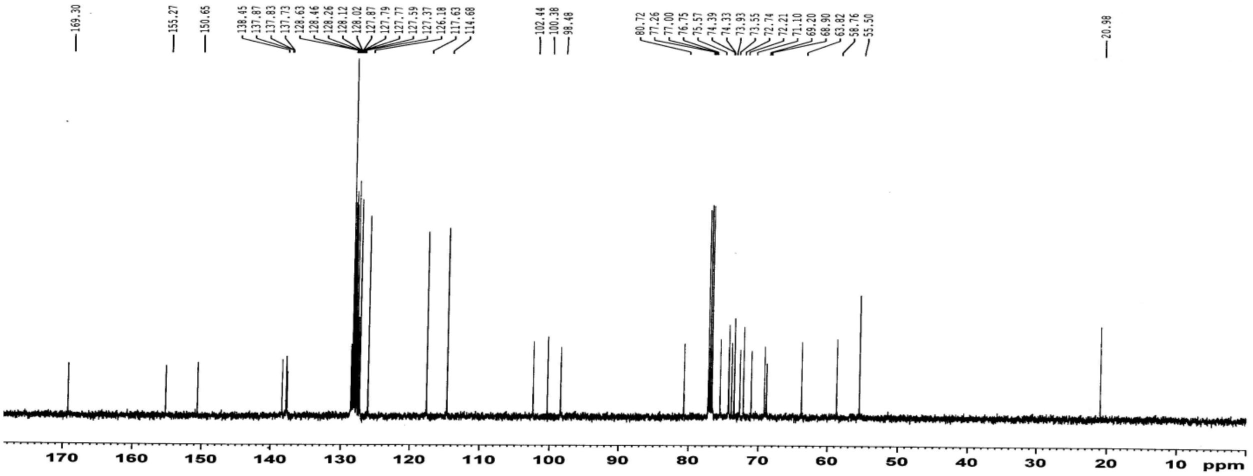

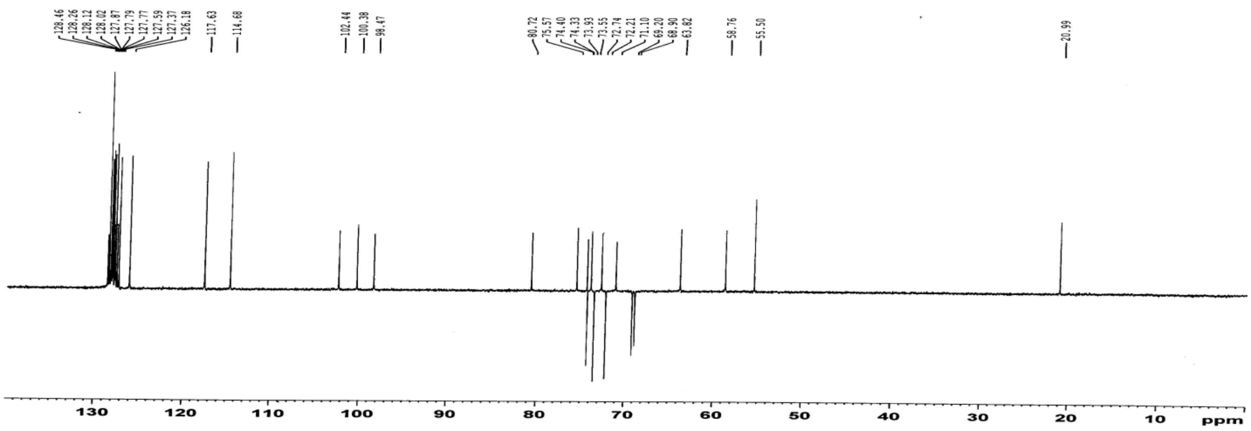

^{1}H, ^{13}C and DEPT 135 NMR spectra of *p*-methoxyphenyl (2-*O*-acetyl-3,4,6-tri-*O*-benzyl-β-D-galactopyranosyl)-(1→3)-2-azido-4,6-*O*-benzylidene-2-deoxy-α-D-galactopyranoside (**8**) ($CDCl_3$).

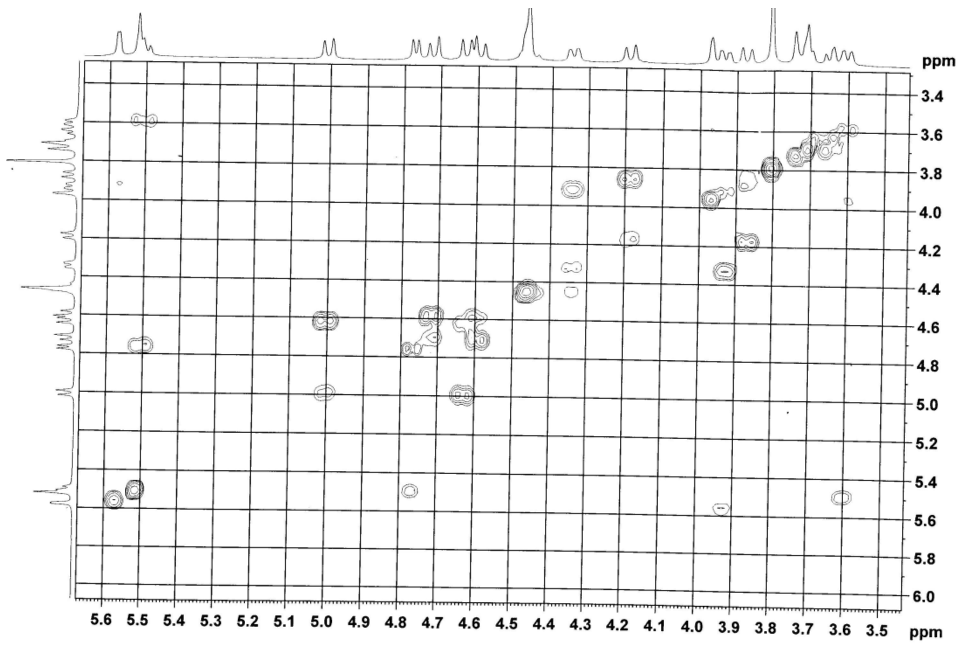

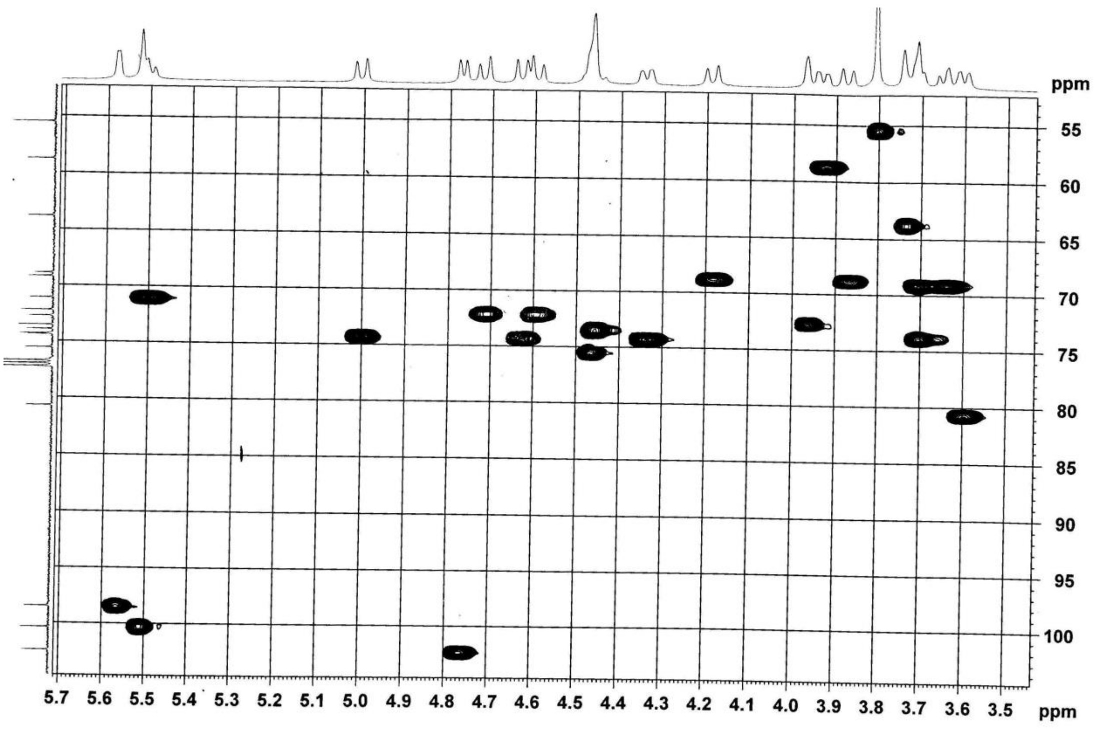

2D COSY and HSQC NMR spectra of *p*-methoxyphenyl (2-*O*-acetyl-3,4,6-tri-*O*-benzyl-β-D-galactopyranosyl)-(1→3)-2-azido-4,6-*O*-benzylidene-2-deoxy-α-D-galactopyranoside (**8**) ($CDCl_3$).

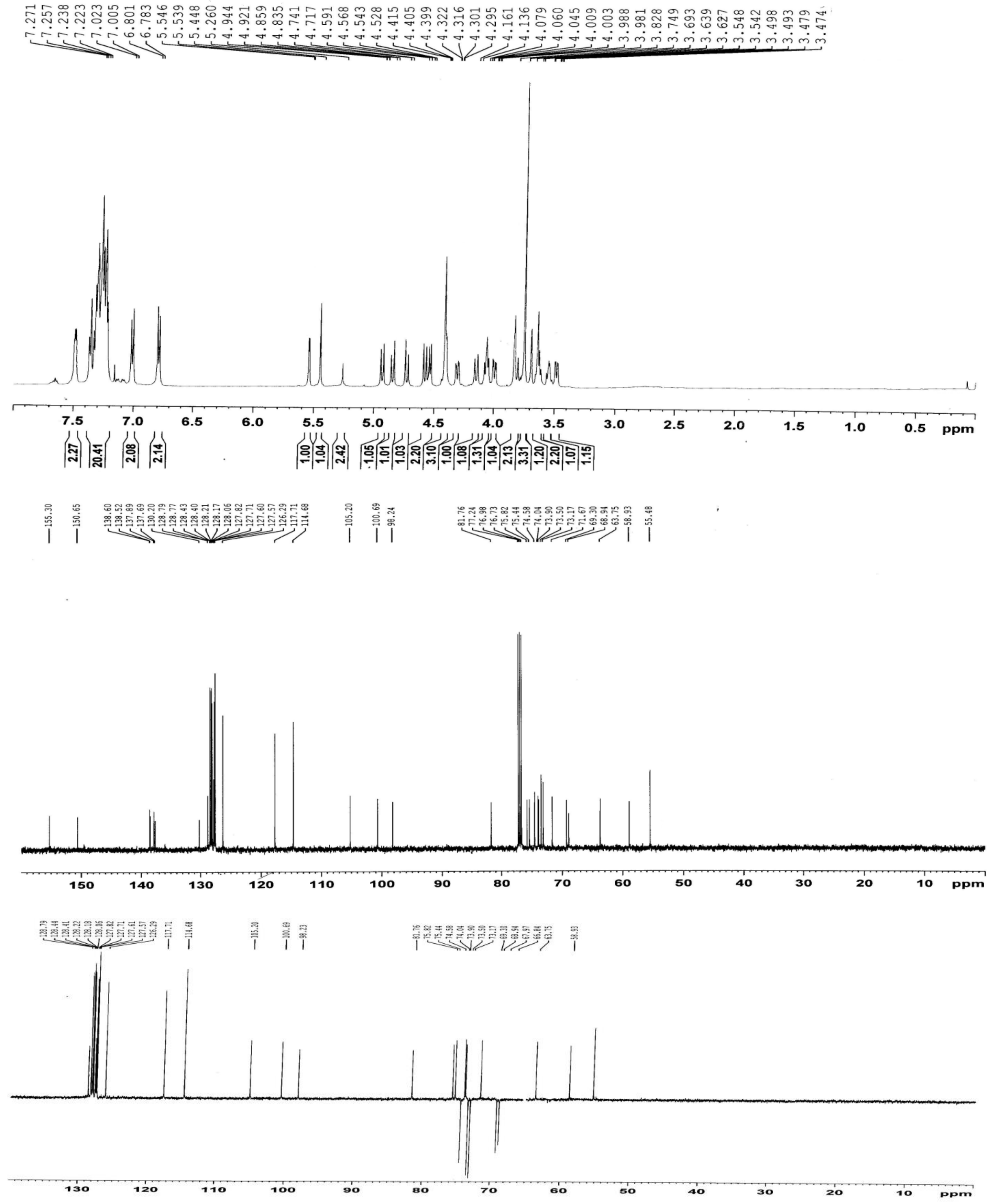

^{1}H, ^{13}C and DEPT 135 NMR spectra of *p*-methoxyphenyl (3,4,6-tri-*O*-benzyl-β-D-galactopyranosyl)-(1→3)-2-azido-4,6-*O*-benzylidene-2-deoxy-α-D-galactopyranoside (**9**) ($CDCl_3$).

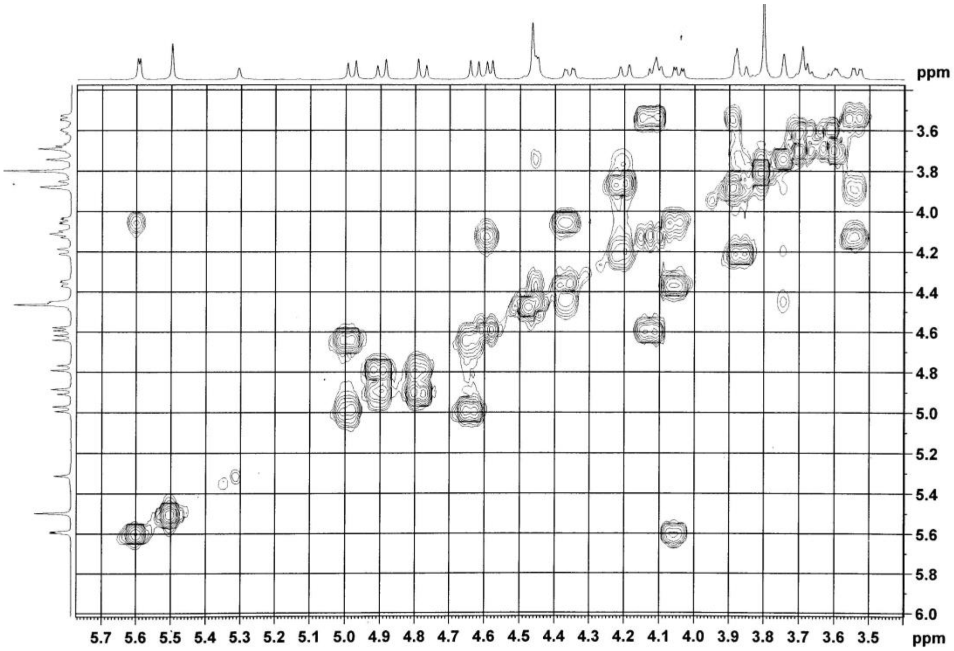

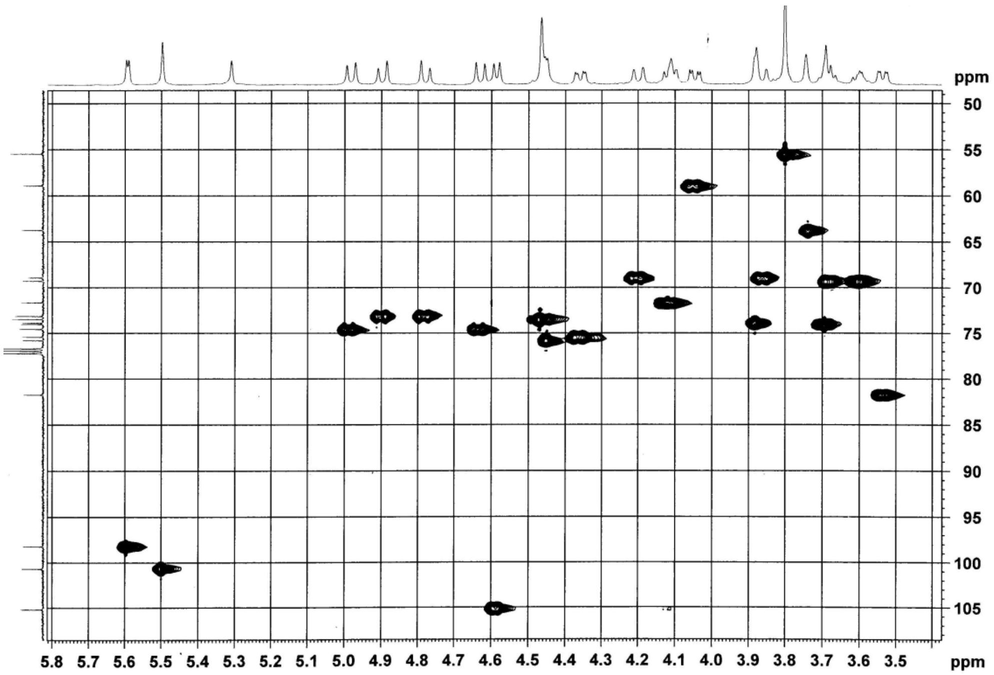

2D COSY and HSQC NMR spectra of *p*-methoxyphenyl (3,4,6-tri-*O*-benzyl-β-D-galactopyranosyl)-(1→3)-2-azido-4,6-*O*-benzylidene-2-deoxy-α-D-galactopyranoside (**9**) ($CDCl_3$) (selected region).

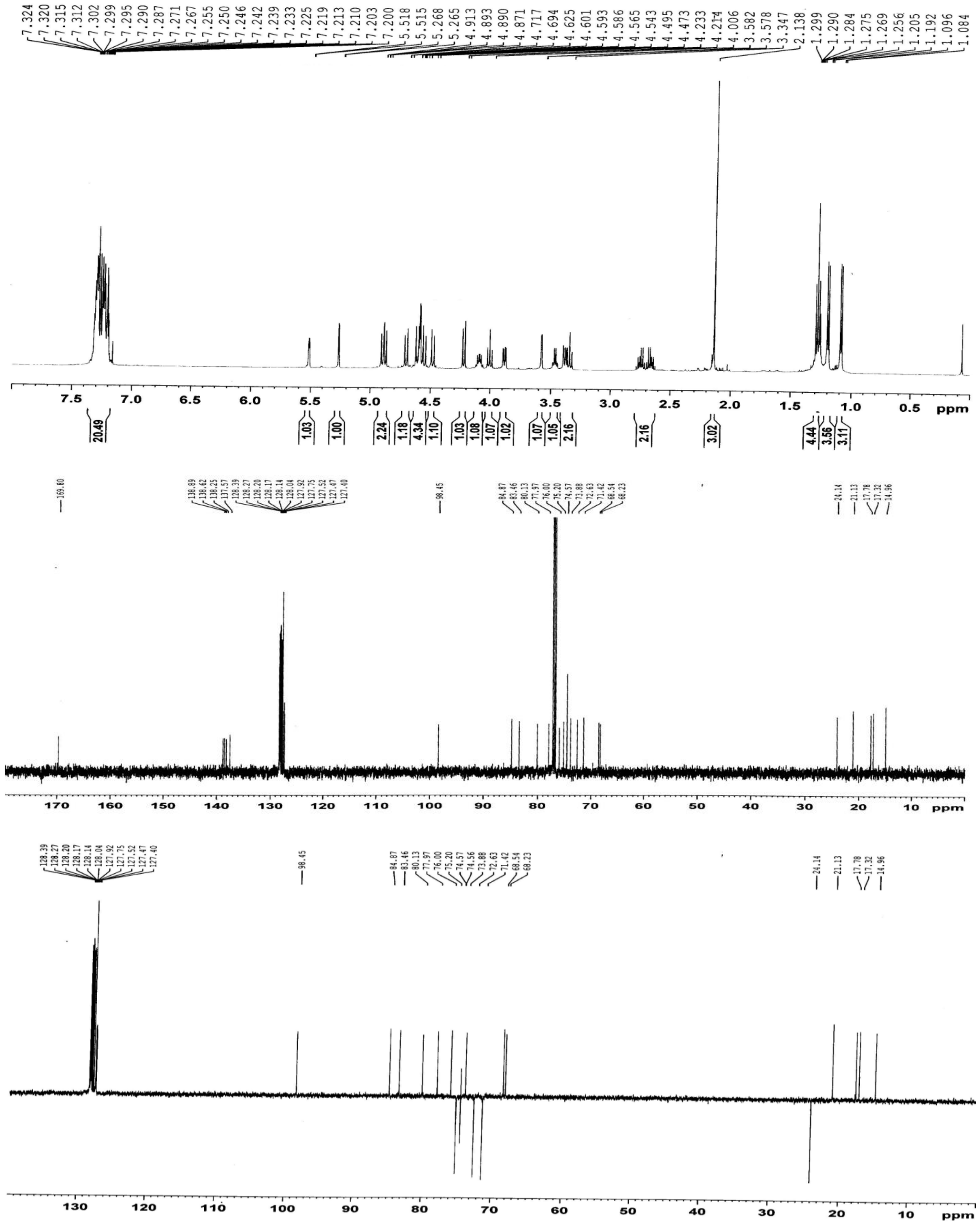

^{1}H, ^{13}C and DEPT 135 NMR spectra of ethyl (2-*O*-acetyl-3,4-di-*O*-benzyl-α-L-rhamnopyranosyl)-(1→2)-3,4-di-*O*-benzyl-1-thio-β-L-fucopyranoside **(10)** ($CDCl_3$).

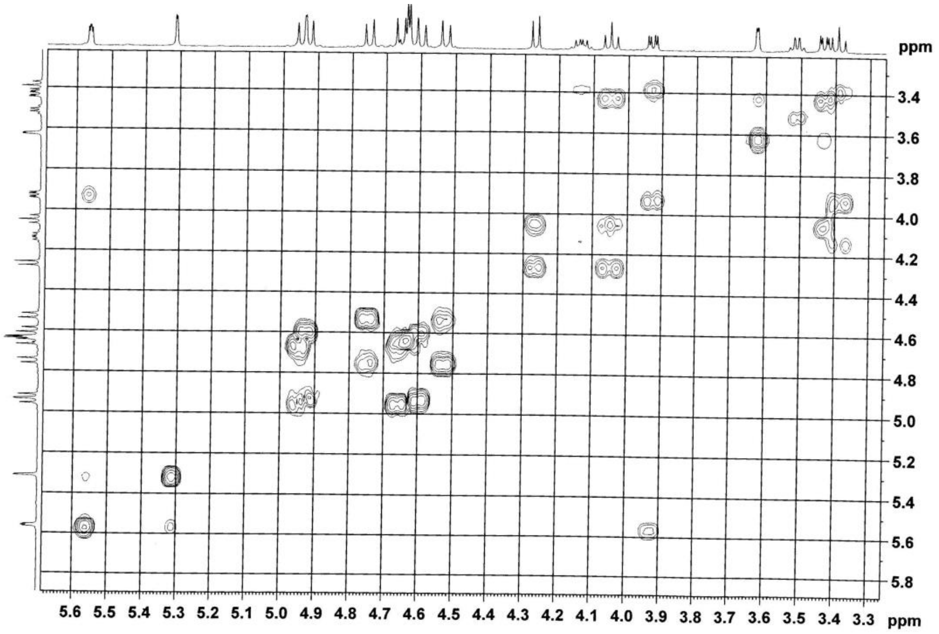

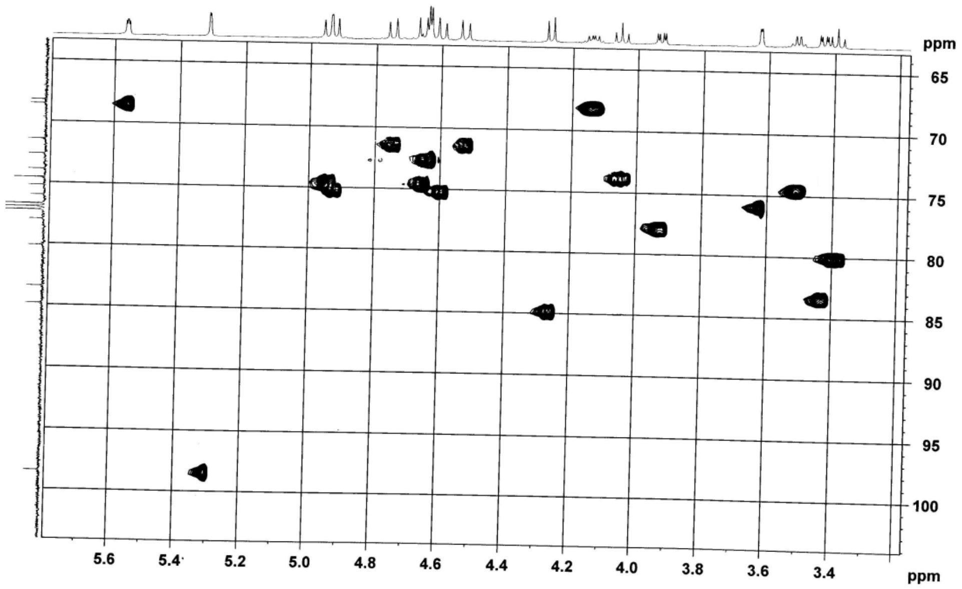

2D COSY and HSQC NMR spectra of ethyl (2-*O*-acetyl-3,4-di-*O*-benzyl-α-L-rhamnopyranosyl)-(1→2)-3,4-di-*O*-benzyl-1-thio-β-L-fucopyranoside **(10)** ($CDCl_3$) (Selected region).

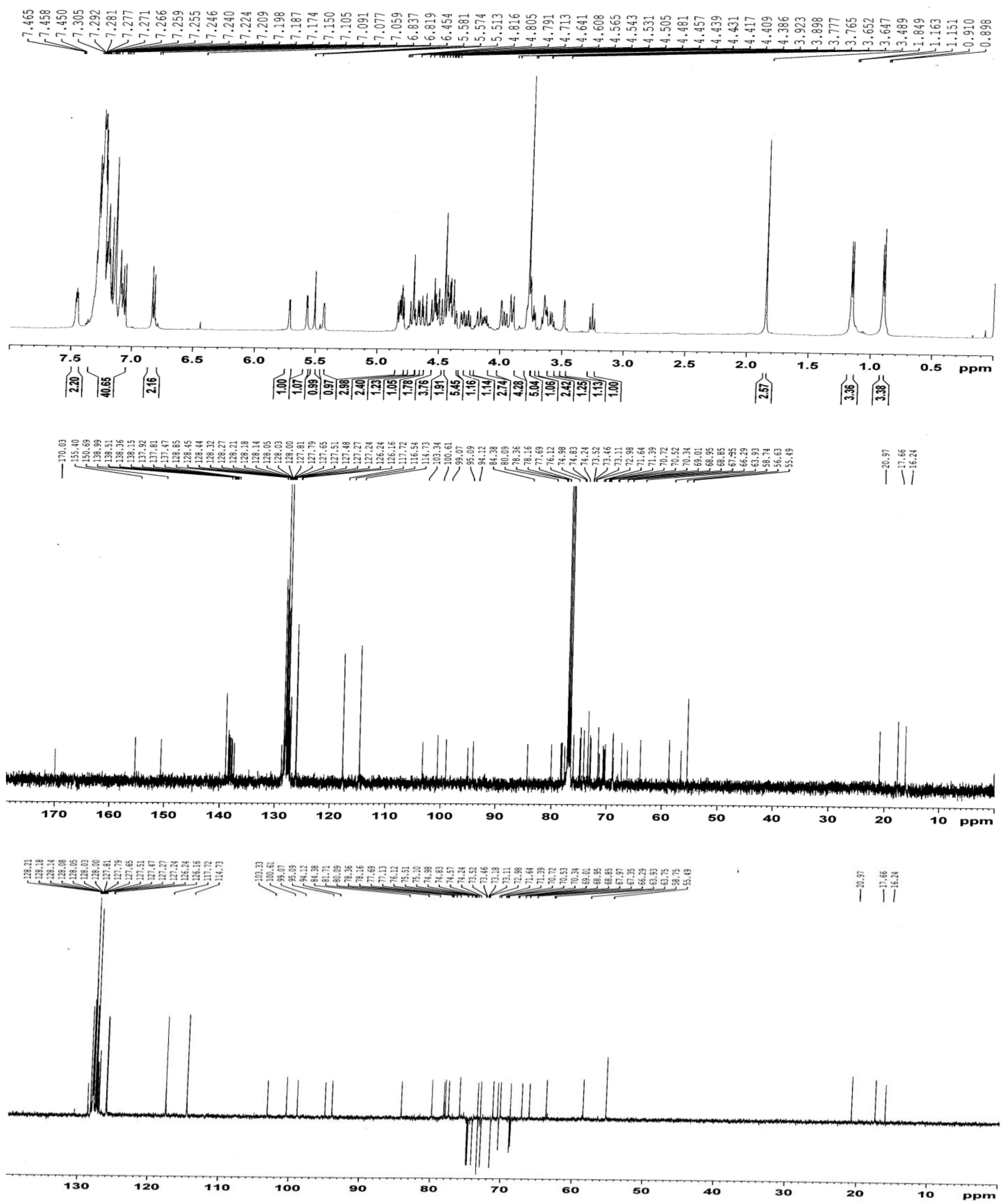

^{1}H, ^{13}C and DEPT 135 NMR spectra of *p*-methoxyphenyl (2-*O*-acetyl-3,4-di-*O*-benzyl-α-L-rhamnopyranosyl)-(1→2)-(3,4-di-*O*-benzyl-α-L-fucopyranosyl)-(1→2)-(3,4,6-tri-*O*-benzyl-β-D-galactopyranosyl)-(1→3)-2-azido-4,6-*O*-benzylidene-2-deoxy-α-D-galactopyranoside **(11)** ($CDCl_3$).

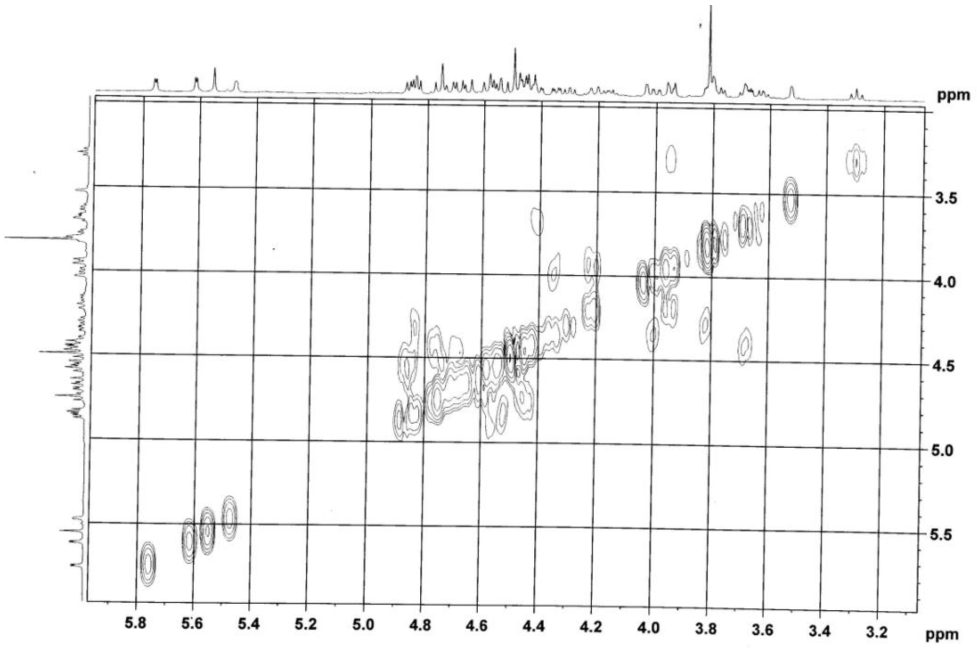

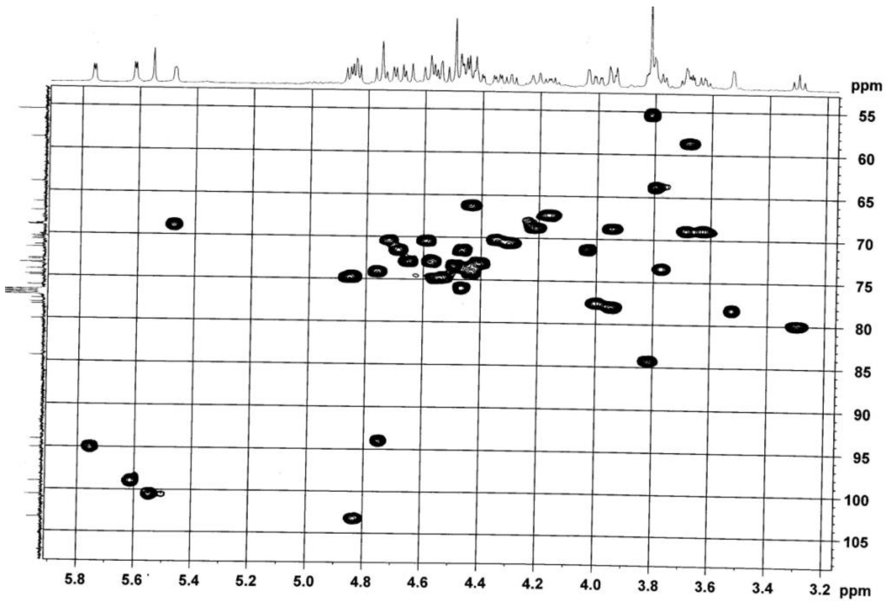

2D COSY and HSQC NMR spectra of *p*-methoxyphenyl (2-*O*-acetyl-3,4-di-*O*-benzyl-α-L-rhamnopyranosyl)-(1→2)-(3,4-di-*O*-benzyl-α-L-fucopyranosyl)-(1→2)-(3,4,6-tri-*O*-benzyl-β-D-galactopyranosyl)-(1→3)-2-azido-4,6-*O*-benzylidene-2-deoxy-α-D-galactopyranoside **(11)** ($CDCl_3$) (Selected region).

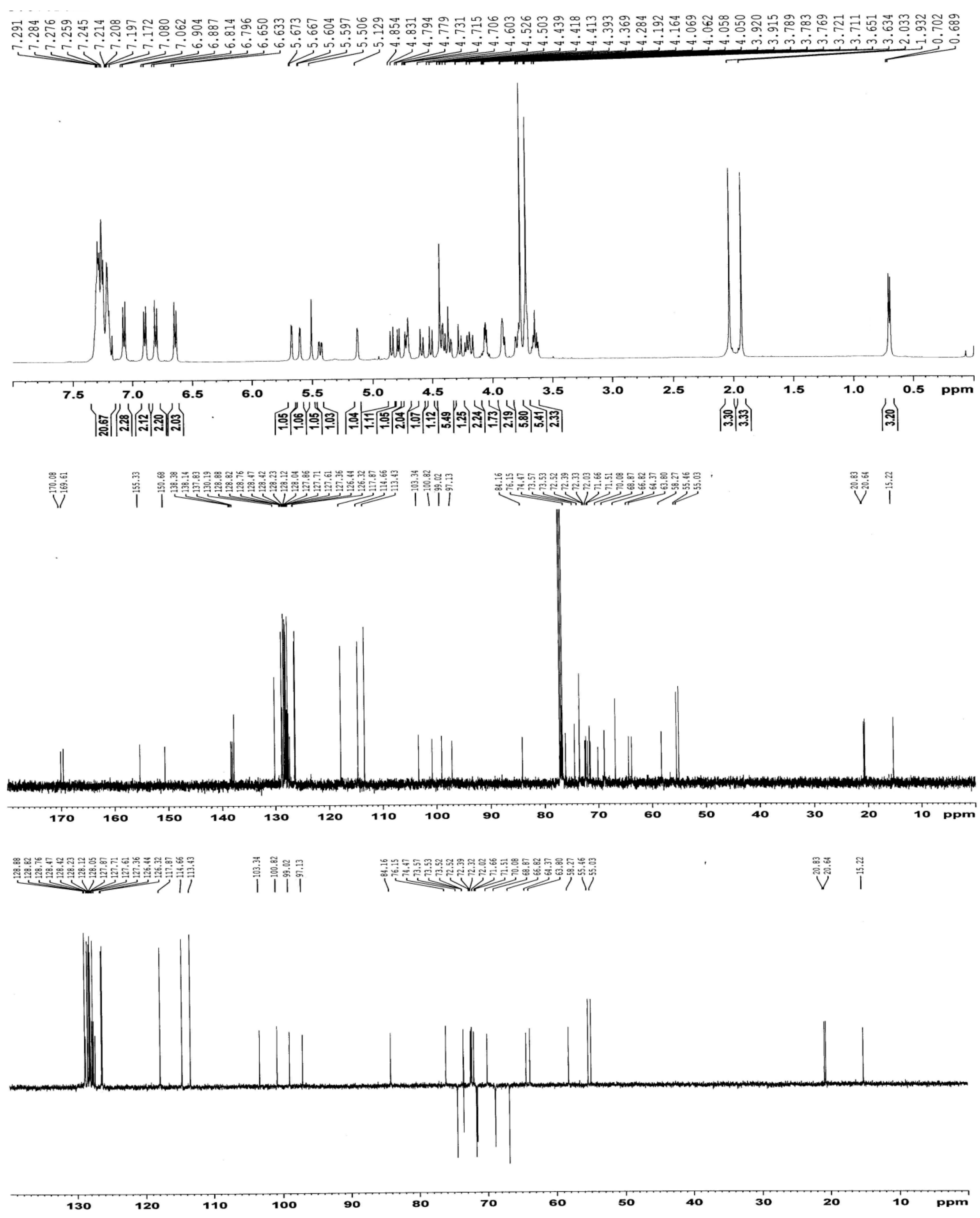

^{1}H, ^{13}C and DEPT 135 NMR spectra of *p*-methoxyphenyl (3,4-di-*O*-acetyl-2-*O*-(*p*-methoxybenzyl))-α-L-fucopyranosyl)-(1→2)-(3,4,6-tri-*O*-benzyl-β-D-galactopyranosyl)-(1→3)-2-azido-4,6-*O*-benzylidene-2-deoxy-α-D-galactopyranoside (**12**) ($CDCl_3$).

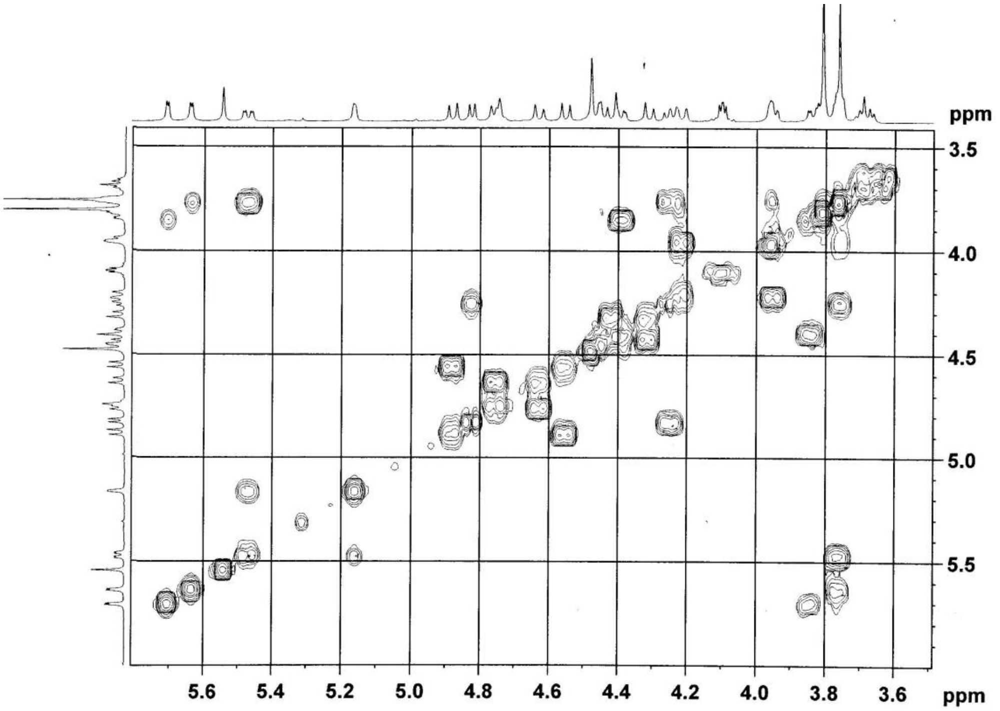

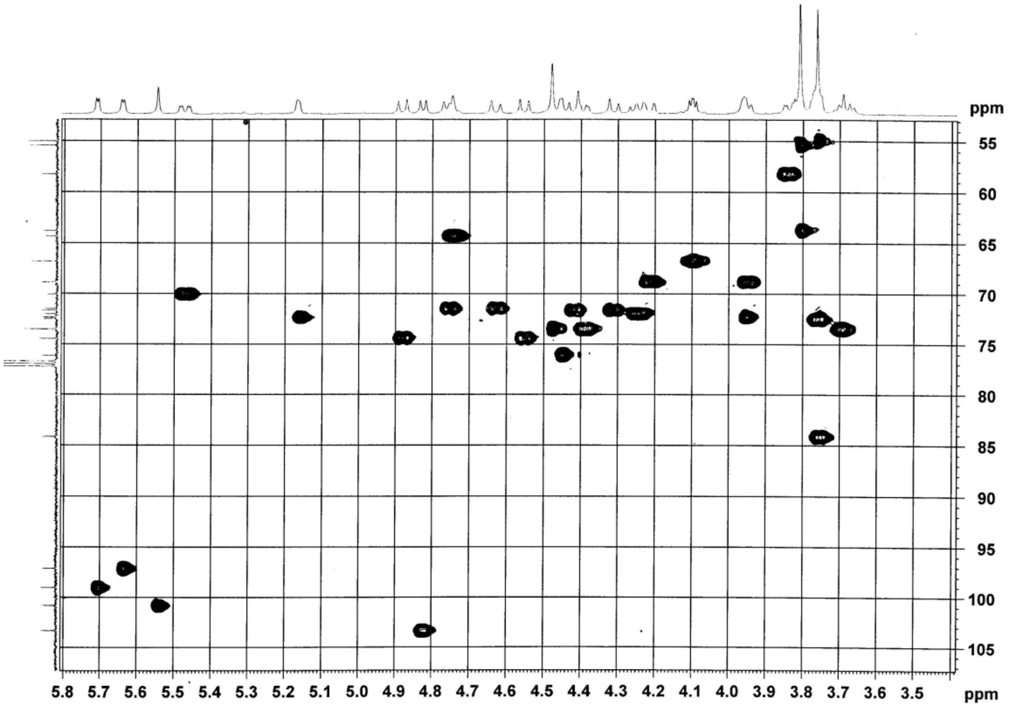

2D COSY and HSQC NMR spectra of *p*-methoxyphenyl (3,4-di-*O*-acetyl-2-*O*-(*p*-methoxybenzyl))-α-L-fucopyranosyl)-(1→2)-(3,4,6-tri-*O*-benzyl-β-D-galactopyranosyl)-(1→3)-2-azido-4,6-*O*-benzylidene-2-deoxy-α-D-galactopyranoside (**12**) ($CDCl_3$) (selected region).

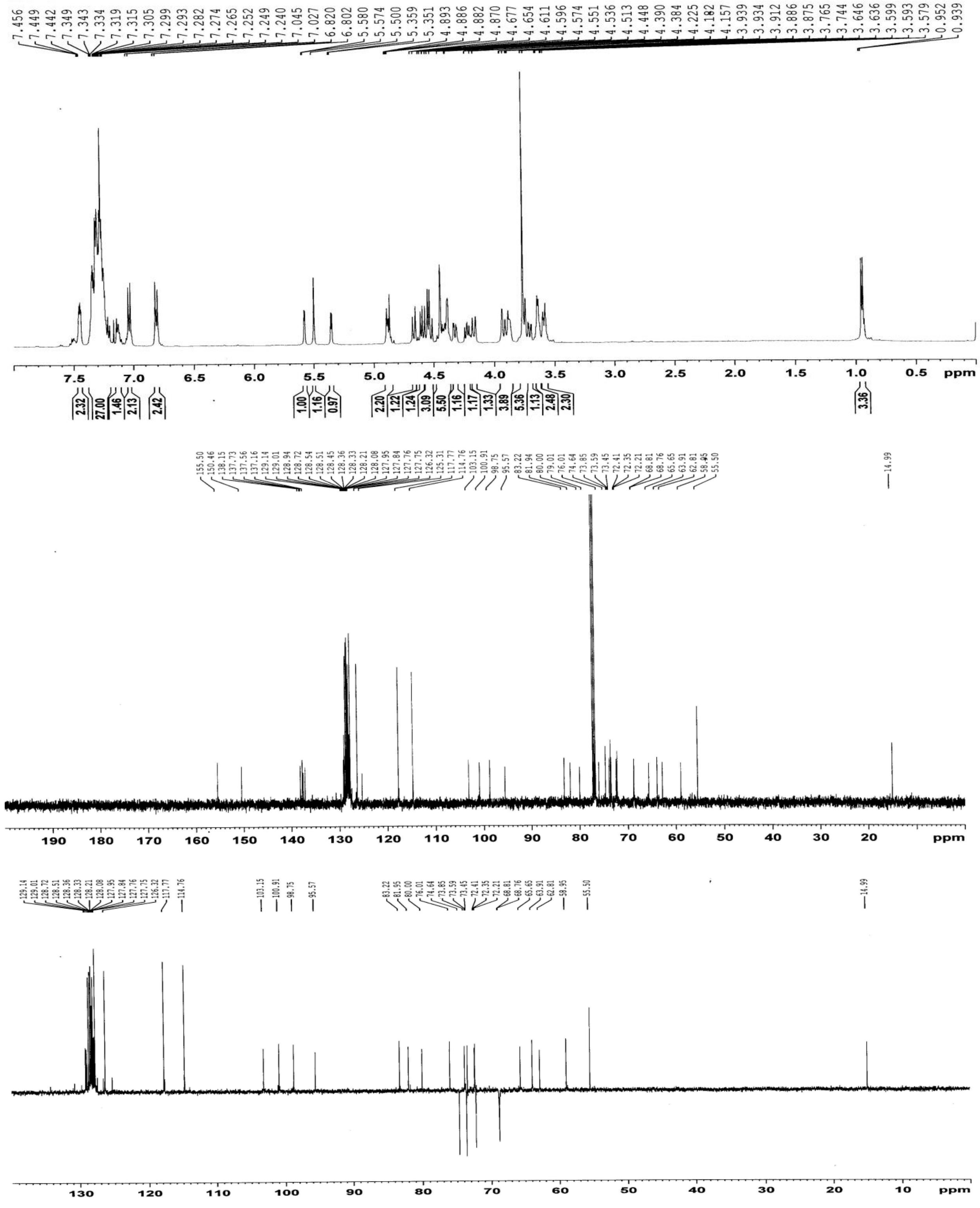

^{1}H, ^{13}C and DEPT 135 NMR spectra of *p*-methoxyphenyl (3,4-di-*O*-benzyl-α-L-fucopyranosyl)-(1→2)-(3,4,6-tri-*O*-benzyl-β-D-galactopyranosyl)-(1→3)-2-azido-4,6-*O*-benzylidene-2-deoxy-α-D-galactopyranoside (**13**) ($CDCl_3$).

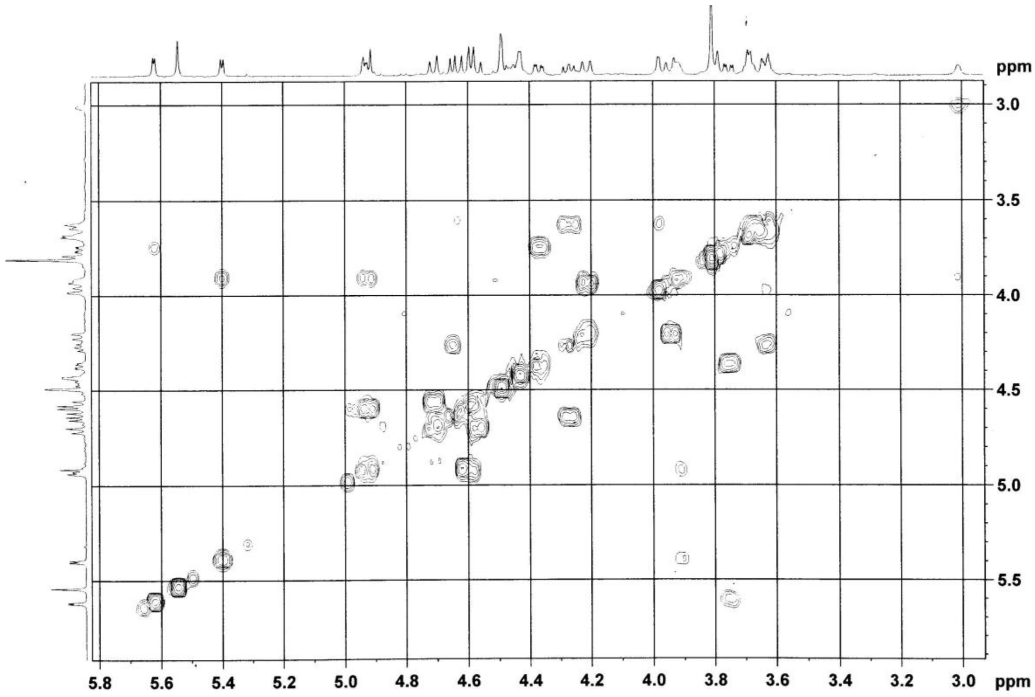

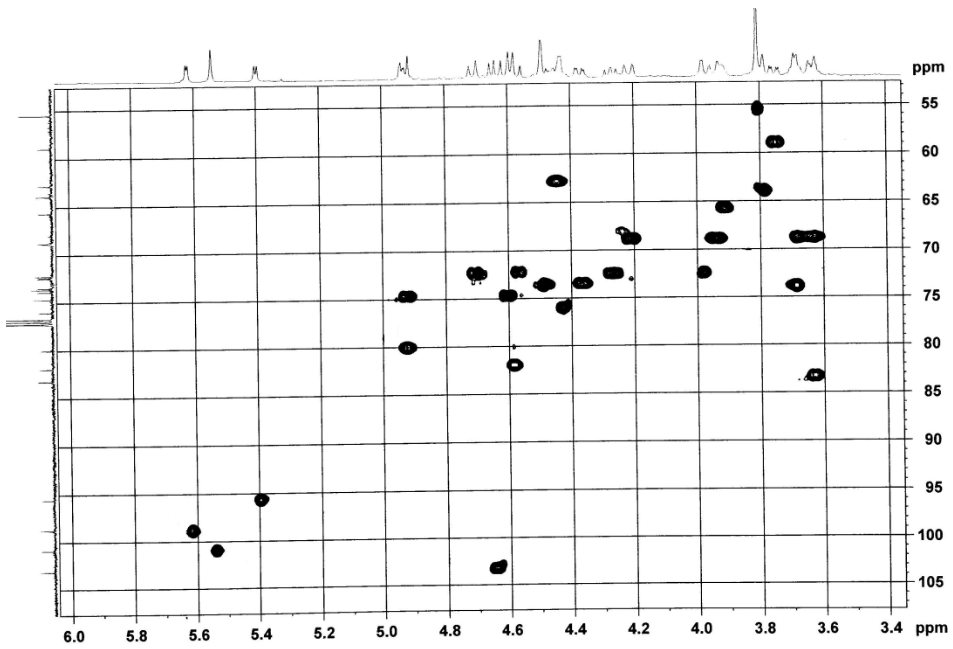

2D COSY and HSQC NMR spectra of *p*-methoxyphenyl (3,4-di-*O*-benzyl-α-L-fucopyranosyl)-(1→2)-(3,4,6-tri-*O*-benzyl-β-D-galactopyranosyl)-(1→3)-2-azido-4,6-*O*-benzylidene-2-deoxy-α-D-galactopyranoside (**13**) ($CDCl_3$) (Selected region).

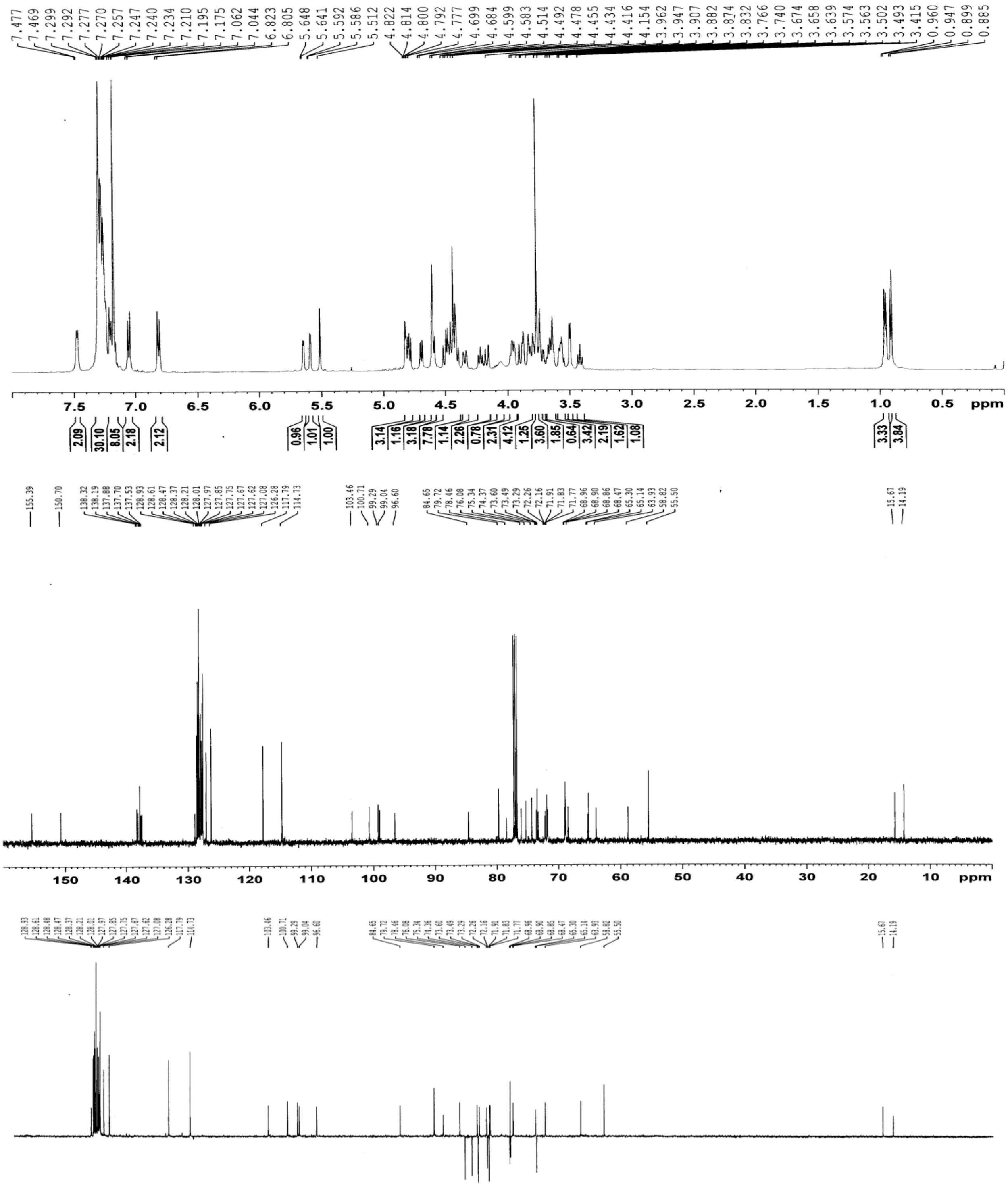

^{1}H, ^{13}C and DEPT 135 NMR spectra of *p*-methoxyphenyl (3,4-di-*O*-benzyl-α-L-rhamnopyranosyl)-(1→2)-(3,4-di-*O*-benzyl-α-L-fucopyranosyl)-(1→2)-(3,4,6-tri-*O*-benzyl-β-D-galactopyranosyl)-(1→3)-2-azido-4,6-*O*-benzylidene-2-deoxy-α-D-galactopyranoside (**14**) ($CDCl_3$).

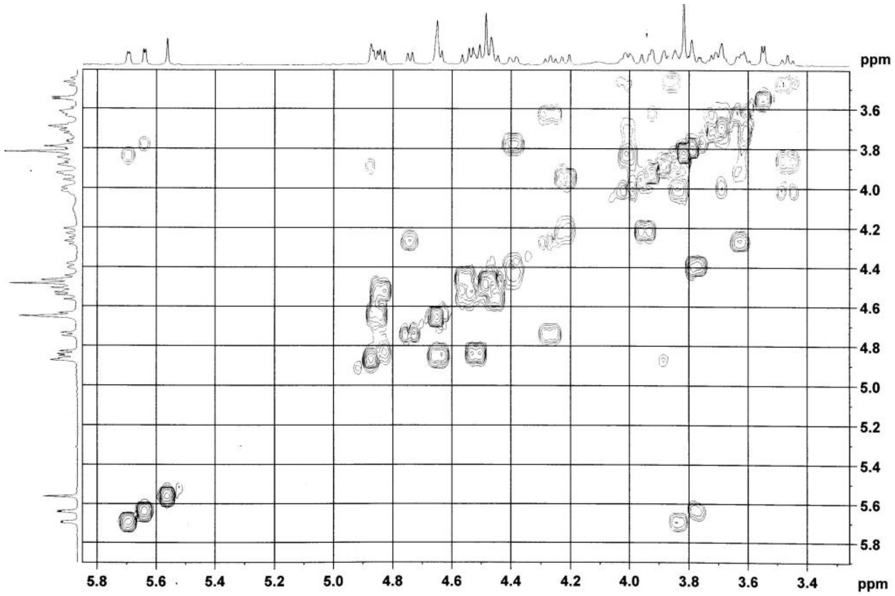

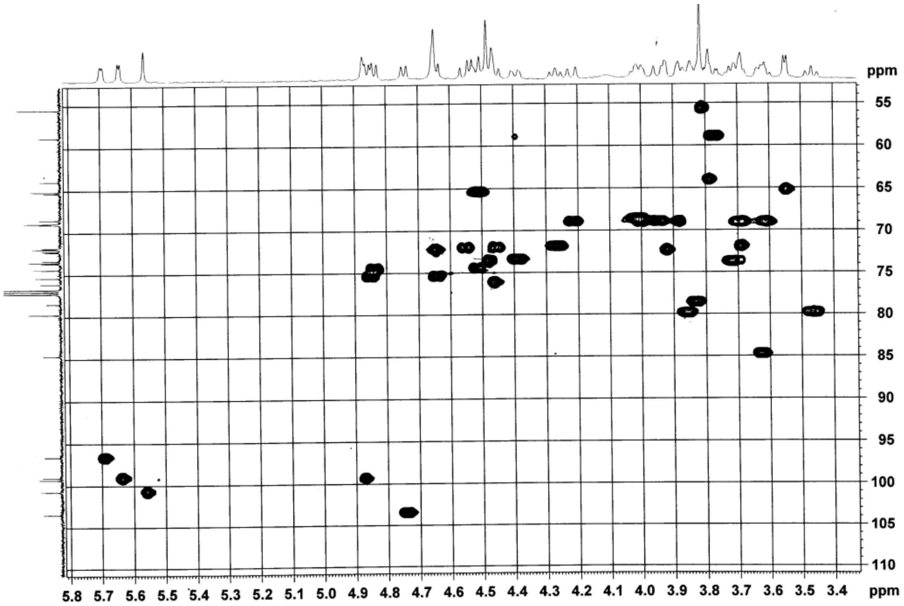

2D COSY and HSQC NMR spectra of *p*-methoxyphenyl (3,4-di-*O*-benzyl-α-L-rhamnopyranosyl)-(1→2)-(3,4-di-*O*-benzyl-α-L-fucopyranosyl)-(1→2)-(3,4,6-tri-*O*-benzyl-β-D-galactopyranosyl)-(1→3)-2-azido-4,6-*O*-benzylidene-2-deoxy-α-D-galactopyranoside (**14**) ($CDCl_3$) (Selected region).

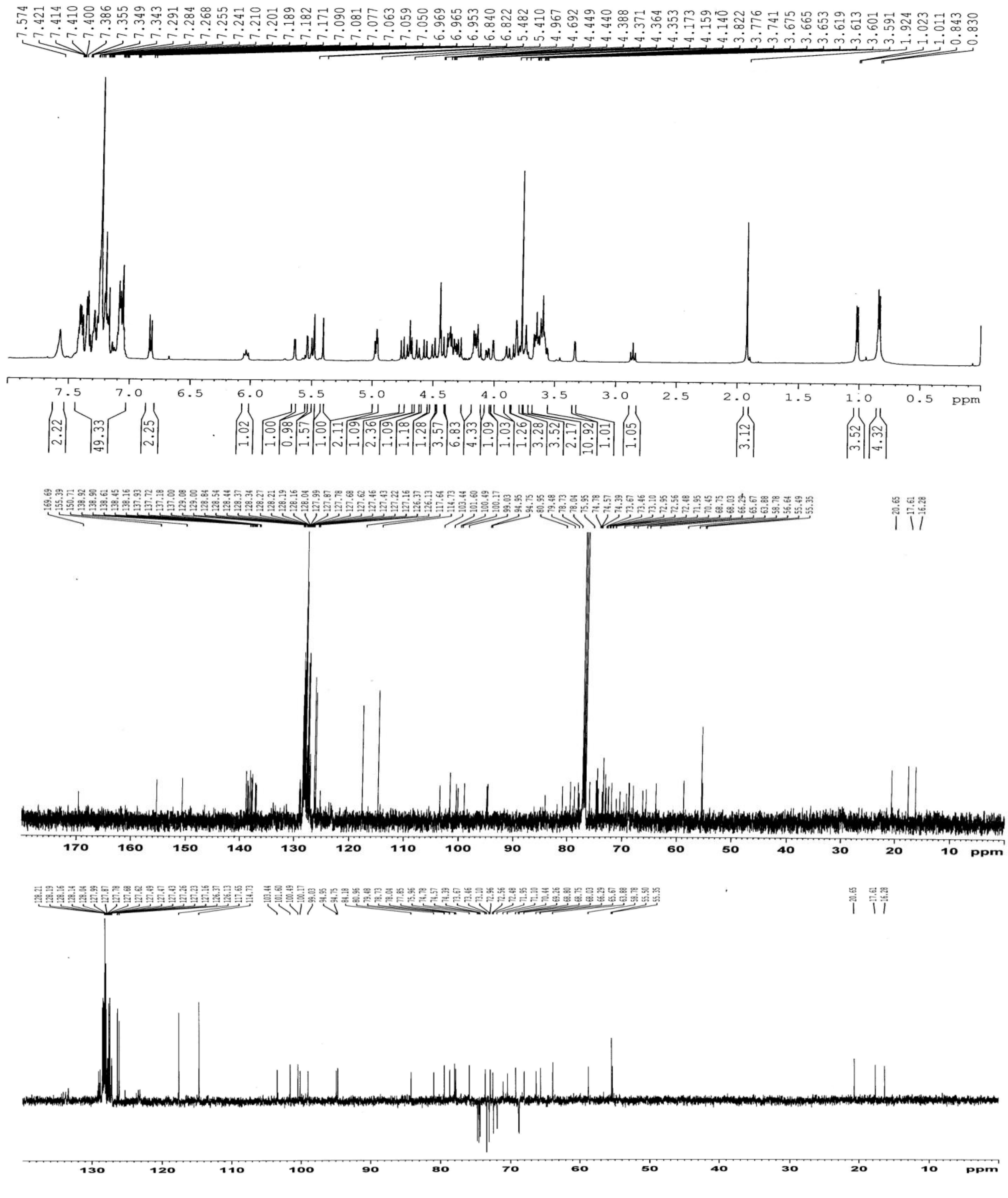

^{1}H, ^{13}C and DEPT 135 NMR spectra of *p*-methoxyphenyl (2-*O*-acetyl-4,6-*O*-benzylidene-2-deoxy-2-*N*-phthalimido-β-D-glucopyranosyl)-(1→2)-(3,4-di-*O*-benzyl-α-L-rhamnopyranosyl)-(1→2)-(3,4-di-*O*-benzyl-α-L-fucopyranosyl)-(1→2)-(3,4,6-tri-*O*-benzyl-β-D-galactopyranosyl)-(1→3)-2-azido-4,6-*O*-benzylidene-2-deoxy-α-D-galactopyranoside (**15**) ($CDCl_3$).

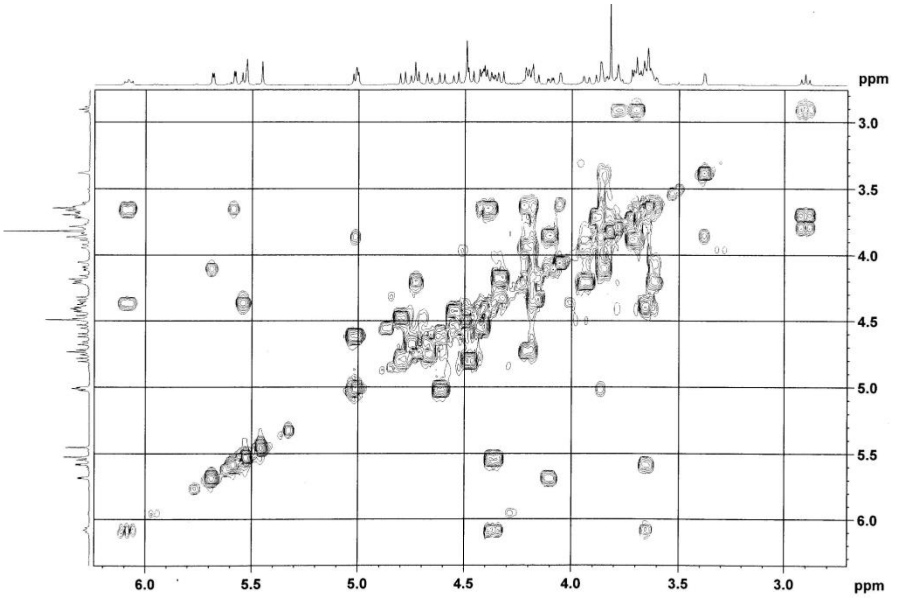

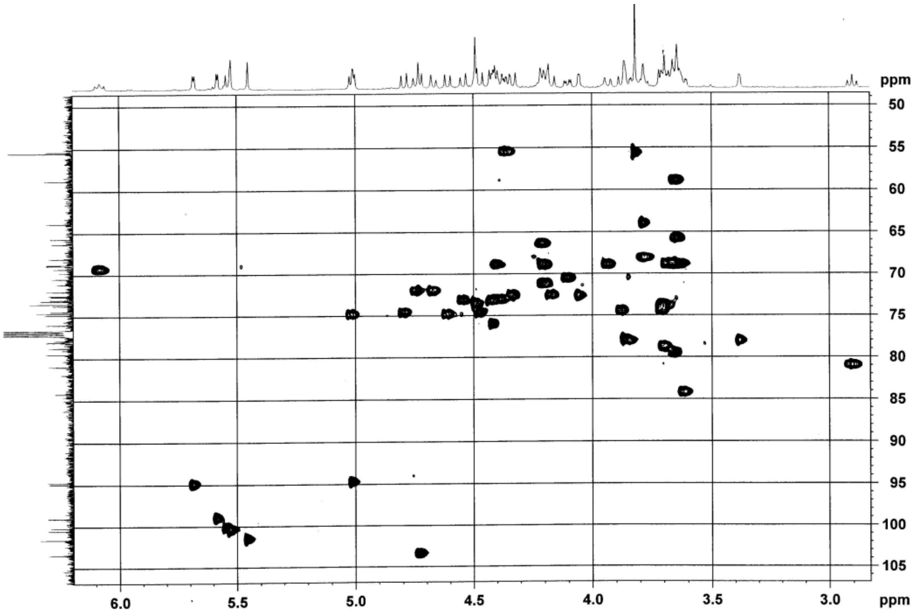

2D COSY and HSQC NMR spectra of *p*-methoxyphenyl (2-*O*-acetyl-4,6-*O*-benzylidene-2-deoxy-2-*N*-phthalimido-β-D-glucopyranosyl)-(1→2)-(3,4-di-*O*-benzyl-α-L-rhamnopyranosyl)-(1→2)-(3,4-di-*O*-benzyl-α-L-fucopyranosyl)-(1→2)-(3,4,6-tri-*O*-benzyl-β-D-galactopyranosyl)-(1→3)-2-azido-4,6-*O*-benzylidene-2-deoxy-α-D-galactopyranoside (**15**) ($CDCl_3$) (selected region).
